# Exogenous loading of extracellular vesicles, virus-like particles, and lentiviral vectors with supercharged proteins

**DOI:** 10.1038/s42003-022-03440-7

**Published:** 2022-05-19

**Authors:** Koen Breyne, Stefano Ughetto, David Rufino-Ramos, Shadi Mahjoum, Emily A. Grandell, Luís P. de Almeida, Xandra O. Breakefield

**Affiliations:** 1 Molecular Neurogenetics Unit, Massachusetts General Hospital - Harvard Medical School, 13th Street, Building 149, Charlestown, MA 02129 USA; 2 Department of Oncology, University of Turin, 10060 Candiolo (TO), Italy; 3 Center for Neuroscience and Cell Biology, Faculty of Pharmacy, University of Coimbra, Coimbra, Portugal

**Keywords:** Cell biology, Gene delivery, Nanoparticles

## Abstract

Cell membrane-based biovesicles (BVs) are important candidate drug delivery vehicles and comprise extracellular vesicles, virus-like particles, and lentiviral vectors. Here, we introduce a non-enzymatic assembly of purified BVs, supercharged proteins, and plasmid DNA called pDNA-scBVs. This multicomponent vehicle results from the interaction of negative sugar moieties on BVs and supercharged proteins that contain positively charged amino acids on their surface to enhance their affinity for pDNA. pDNA-scBVs were demonstrated to mediate floxed reporter activation in culture by delivering a Cre transgene. We introduced pDNA-scBVs containing both a CRE-encoding plasmid and a BV-packaged floxed reporter into the brains of Ai9 mice. Successful delivery of both payloads by pDNA-scBVs was confirmed with reporter signal in the striatal brain region. Overall, we developed a more efficient method to load isolated BVs with cargo that functionally modified recipient cells. Augmenting the natural properties of BVs opens avenues for adoptive extracellular interventions using therapeutic loaded cargo.

## Abstract (web summary)

The development of positive supercharged protein loading of cell membrane-based biovesicles, including extracellular vesicles, virus-like particles, and lentiviral vectors, as a strategy to deliver exogenous plasmid DNA to target cells in vitro and in vivo is presented.

## Introduction

Cell-derived biovesicles (BVs) comprise a large group of bioentities present in the extracellular space ranging from extracellular vesicles (EVs), including exosomes and microvesicles^1^, viral-like particles to enveloped viruses^2^, including lentiviral vectors (LVVs). The common elements among all these BV types are their natural capacity to incorporate biomolecules (endogenous cargo or endocargo) from a parent cell, their subcellular scale (nano to micro), their release into the extracellular space, and protection of their luminal contents by a lipid membrane^3,4^.

Non-infectious EVs have gained more and more attention as a potential therapeutic-delivery vehicle option^5–7^. EVs have the potential to counter major drawbacks of virus-based vectors, such as immunogenicity, potentially allele-disruptive genome integration, small capacity for additional non-viral encoded biocargo, and potential cytotoxicity. Biomarker studies have demonstrated that the host tolerates high numbers of EVs produced by virtually every cell in the body in biofluids and extracellular spaces, including both from diseased and normal tissues^8^. Limiting factors in EV therapeutic development are our understanding of defined mechanisms of biomolecule sorting from the source cells into EVs and their fate in recipient cells^3,9^. The bioactivity of EV cargo acting upon recipient cells has been the subject of debate in recent years, due in part to experimental limitations at the single EV level^10^ and quantitation of functional cargo^11^. The overall observed effect of the EV endocargo in recipient cells is seemingly low and might need additional boost signals to improve therapeutic relevance or multiple EV exposures.

Here, we explore a strategy that is not limited by the donor cell’s ability to package a desired payload into EVs. This avenue is called exogenous EV loading, which others have pursued for loading synthetic RNAs and proteins with lipofection agents or electroporation^12,13^. Our strategy exploits synthetically reprogrammed proteins with positively charged amino acids, such as arginine and lysine, exposed on the outside of the protein structure, while their functional amino acids remain unchanged from their parental structure^14^. The biophysical properties of these positively supercharged proteins (+scProteins) enable association with and migration through the cell membranes of living cells^15^. Here we demonstrate that +scProteins utilizes EV properties to load negatively charged DNA species and aid in the functional delivery of the latter.

## Results

### Exogenous loading of extracellular vesicles with positive supercharged proteins

**Fig. 1:**
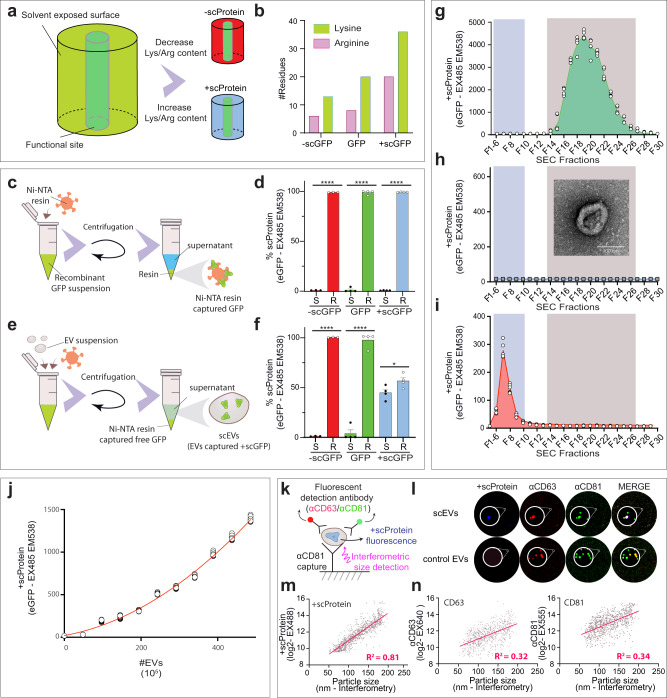
The number of Lys/Arg surface residues are key for a protein’s potential to associate with extracellular vesicles. **a** Protein surface mutagenesis. Proteins can be genetically reengineered to display different numbers of lysine (Lys) or arginine (Arg) residues on the solvent exposed surface. Green fluorescent protein (GFP) with reduced or increased levels of Lys/Arg residues on the surface are called negative supercharged GFP (-scGFP) or positive supercharged GFP (+scGFP), respectively. **b** Arginine/Lysine content in supercharged proteins (scProteins). Number of Lys and Arg incorporated in the supercharged GFP proteins are indicated in green and pink bars, respectively. **c** Recombinant supercharged protein pull-down. Ni-NTA resin pull-down of free recombinant +scGFP, GFP or, −scGFP to deplete supernatant of GFP fluorescence. **d** In extracellular vesicles (EV)-free solutions, GFP fluorescence was compared between supernatant (S) after incubation with recombinant −scGFP (*n* = 3), GFP (*n* = 4), or +scGFP (*n* = 4) proteins and the Ni-NTA resin pellet (R). Data are represented in percentage whereby the total fluorescence of the supernatant (S) and Ni-NTA resin pellet (R) represents 100 percent. **e** Test to verify assembly of GFP with EVs. Interaction of recombinant GFP proteins with EVs was investigated through comparing fluorescent levels of EV-associated vs -unassociated free recombinant protein post-GFP affinity pull-down with Ni-NTA resin. **f** +scProtein generates supercharged EVs (scEVs). Post-incubation of HEK293T EVs with −scGFP (*n* = 3), GFP (*n* = 4) or +scGFP (*n* = 4), we added Ni-NTA resin to evaluate whether GFP loaded HEK293T EVs would remain in the supernatant as scEVs. GFP fluorescence was quantified in both supernatant (S) and resin (R). Data are represented in percentage whereby the total fluorescence of the supernatant (S) and Ni-NTA resin pellet (R) represents 100 percent. **g** Size exclusion chromatography (SEC) profile of fluorescent recombinant +scGFP. Profile when resolving recombinant +scGFP (*n* = 6) with SEC. A fluorescent signal was detected in the late protein fractions (F) i.e., F14 to F28. Graph represents GFP fluorescence in each SEC fraction. The blue band represents the SEC fractions expected to contain EVs and the grey band represents the SEC fractions expected to contain free proteins. **h** SEC profile of non-fluorescent SEC-purified EVs. Non-fluorescent HEK293T EVs were not detectable (*n* = 15). Graph represents GFP fluorescence in each SEC fraction. The blue band represents the SEC fractions expected to contain EVs and the grey band represents the SEC fractions expected to contain free proteins. A transmission electron microscopic image of a non-fluorescent EV used for +scProtein loading is shown. Scalebar = 100 nm. **i** SEC profile of scEVs post-Ni-NTA cleanup. HEK293T scEVs (*n* = 6) were generated through combining +scGFP, non-fluorescent HEK293T EVs, and Ni-NTA cleanup. scEVs had a distinct SEC profile whereby GFP fluorescence was observed in the lower SEC fractions, i.e., F7–F12, showing incorporation into EVs. The blue band represents the SEC fractions expected to contain EVs and the grey band represents the SEC fractions expected to contain free proteins. **j** Correlation of +scProtein content and EV number in scEV solution. +scProtein loading into EVs was dependent on the number of SEC-purified EVs in solution. Red line demonstrates the non-linear correlation (R2 = 0.9934) between +scProtein fluorescence and the number of 10^5 HEK293T EVs (*n* = 16) in a scEVs solution. **k** Single scEV analysis with Exoview. Schematic of Exoview method that measures +scProtein and tetraspanin content, as well as particle size on single scEVs. HEK293T scEVs were captured by CD81 antibodies and detected with CD81 and CD63 antibodies in green and red channels, respectively. +scProtein detection occurred in the blue channel. Fluorescent intensities are used to quantify CD63, CD81, and +scProtein levels while interferometric measurements informed about the size of the scEV. **l** +scProtein fluorescence colocalizes with EV markers in scEVs. These are representative fluorescent images of αCD81 capture spots whereby we identify +scProteins (blue), CD63 (red), CD81 (green) in loaded (scEVs) and unloaded (control EVs) from HEK293T EV samples. Overlap of all markers is visualized in white, overlap of αCD63 and αCD81 is in yellow. **m** Exogenous loaded +scProtein content in scEVs is EV size dependent. In scEVs, we compared particle size measured with interferometry with +scProtein content through fluorescent intensity. The correlation between scEV size and +scProtein fluorescence was R2 = 0.81. The linear model is indicated in red. **n** Endogenous loaded tetraspanin content is not EV size dependent in scEVs. For tetraspanin content in scEVs, we compared tetraspanin fluorescence with particle size. The correlation of particle size and detection antibodies CD63 or CD81 was 0.32 and 0.34, respectively. +scProtein, CD63 and CD81 were detected in scEVs between 50 and 200 nm, with 50 nm being the detection limit of the Exoview method. The linear model is indicated in red. Data are presented with mean and SEM (error bars) and analyzed with unpaired t-test. **** and * represent *p*-values of <0.0001 and <0.5, respectively. R2 represents the statistical measure of how close the data are to the fitted regression line.

Green fluorescent protein (GFP) has a beta barrel scaffold with a centre chromophore^16^. Through site-specific mutagenesis of the centre structure, the fluorescent spectrum of this fluorescent protein can be switched from green emission (EX485-EM538) to cyan emission (EX433-EM475)^17^ (Fig. S1), while site-directed mutagenesis of the exposed surface of GFP alters the biophysical properties of the molecule (Fig. 1a). The net charge of GFP can be decreased or increased by modulation of the lysine/arginine ratio^18^, generating a negatively or a positively supercharged protein (scProtein) (Fig. 1b). To evaluate the affinity of proteins with different net charges to EVs, three recombinant His-tagged eGFP homologues (−scGFP, eGFP, and +scGFP – amino acid sequence in supplemental Note 1) were generated and evaluated for their binding with His-tag affinity Ni-NTA resin (Fig. 1c). The latter was observed to capture 99.1 ± 0.13, 98.8 ± 2.1, and 99.3 ± 0.19% of detectable fluorescence intensity from suspensions of recombinant −scGFP, eGFP, and +scGFP, respectively (Fig. 1d). However, when −scGFP, eGFP, and +scGFP recombinant proteins were incubated with purified EVs from HEK293T cells (Fig. 1e), 99 ± 0.3, 96.1 ± 7.1, and 55.9 ± 6.8% of the GFP fluorescence was retrieved in the His-tag affinity Ni-NTA resin pellet, respectively (Fig. 1f). We hypothesized that EV association prevented the +scGFP His-tag from binding to Ni-NTA, generating a +scProtein-EV assembly, here called supercharged EVs (scEVs). In essence, the Ni-NTA capture of unloaded free recombinant +scGFP (Fig. S2a-I) allows us to generate a fluorescent scEV suspension (Fig. S2a-II), whereby the +scGFP co-immunoprecipitates with CD63 immuno-affinity beads indicative of +scProtein association with EVs (Fig. S2a-III). We verified this hypothesis by resolving the +scGFP fluorescent suspension before and after Ni-NTA cleanup by size exclusion chromatography (SEC) (Fig. S2b-I). EVs are relatively large particles (30–200 nm)^19^ that peak in the low-numbered SEC fractions (F7-11), while signal retrieved in the later SEC fractions (F13-F28) represent free +scProteins (2–3 nm). This can be demonstrated when resolving a +scGFP solution (without EVs) with SEC. In this case, the fluorescent signal was only detected in the later fractions (Fig. 1g). When +scGFP was mixed with non-fluorescent SEC-purified HEK293T EVs (Fig. 1h) for 45 min prior to SEC, the fluorescence could be detected as two peaks (Fig. S2b-II). The F13-F28 SEC fraction peaking in the latter profile was lacking in a SEC profile post-Ni-NTA cleanup (Fig. 1i and Fig. S2b-III). Thus, confirming our interpretation that Ni-NTA cleanup ensures +scProtein-associated EV purification. Moreover, we explored whether our method also reduced potential aggregate formation in our suspension that might be formed due to mixing of +scGFP with EVs (Fig. S2c). With Nanosight particle analysis of EVs before (Fig. S2c-I) and after +scProtein loading (Fig. S2c-II), a shift in profile was seen from a homogenous one peak profile to a two-peak profile. The Ni-NTA cleanup to generate scEVs excluded aggregates resulting in a profile expected for a single vesicle suspension (Fig. S2c-III). The unique affinity of isolated EVs for +scProteins was further demonstrated by the dependency of +scGFP capture on the number of EVs in solution. When a higher number of SEC-purified HEK293T EVs were provided to the same amount of +scGFP (15 pmol), an increase in +scGFP fluorescence was observed (Fig. 1j). The correlation between the number of scEVs and the increase in fluorescence due to +scGFP association is Y = 20.36 + 0.8214X + 0.004077 X^2^ (R2 = 0.99; *p* < 0.005), where Y represents the scProtein emitted fluorescence and X the number of 10^5^ EVs. A population view of our resulting scEV suspension was investigated with ExoView^®^ analysis^20^ (Fig. 1k). Hereby, scEVs are captured on anti-CD63, anti-CD81, or anti-CD9 printed in distinguishable spots on an ExoView^®^Chip and compared to an isotype IgG control spot. On a capturing antibody spot, a single EV is bound allowing a multiparametic comparison through colocalizing fluorescent signals of +scProtein in the blue channel and two detection antibodies, anti-CD63 in the red channel and anti-CD81 in the green channel (Fig. 1l). Specific scEV binding to our ExoView^®^Chip was confirmed as 130.5 ± 21.9, 361.3 ± 52.5, 126 ± 21.2 fluorescent +scProtein events detected on the anti-CD63, anti-CD81, anti-CD9 capture spots, respectively compared to 7 ± 4.6 events on the IgG control spot (Fig. S3a). We also compared a non-loaded HEK293T EV sample with a HEK293T scEV sample (Fig. S3b). In our scEVs sample, we observed that the sample contained both EVs that associated with +scProtein and EVs that had not, with 8.2% ± 2.0% of the EVs being +scProtein positive. The non-exposed +scProtein exposed EV sample had a similar heatmap profile compared to EVs without +scProtein in our scEV sample. However, EVs with +scProtein in the scEV sample were enriched in an EV subpopulation containing both CD81 and CD63. With interferometric sizing measurements (IM) at each capturing antibody we determined that the level of +scProtein fluorescence is dependent of the size of the loaded EV (Fig. 1m). The linear correlation is log_2_(Y) = 0.03*X + 7.7 (R2 = 0.81; *p* < 0.0001), where Y represents the +scProtein emitted fluorescence and X the size of EVs. The slope of this correlation (slope = 0.03) was higher than when comparing CD81 (slope = 0.019) and CD63 (slope = 0.019) detection antibody emitted fluorescence with IM (Fig. 1n). Also, the R2 value stipulating the strength of the relationship between the fluorescence and the IM was lower for CD81 (R2 = 0.34) and CD63 (R2 = 0.32). Altogether, our data indicates that CD63 and CD81 are incorporated in a similar fashion into EVs by the donor HEK293T cell and that exogenous +scProtein loading is more size dependent compared to endogenous loading of tetraspanins. This observation was confirmed when comparing the EV size indicated by IM (Fig. S3c-I) and anti-CD63 probe fluorescence (Fig. S3c-II) of CD63^+^CD81^+^ events between non-loaded EV samples and loaded scEV samples. Indeed, in the CD63^+^CD81^+^ subpopulation the size of single EVs was larger with +scProtein (70–190 nm) compared to EVs without +scProtein and unloaded control EVs (50–90 nm), while the CD63 fluorescence was the same. The latter observation supports inclusion of single EVs in our analysis. We calculated that 7–10% of the EVs in our +scProtein-exposed EV sample are loaded and 81.8% of the EVs with +scProtein have between 1 and 10 scProteins; 13.6% have between 10 and 20 scProteins; 2.7% have between 20 and 30 scProteins; and 1.8% have more than 30 scProteins (Fig. S3d).

### Extracellular vesicles properties aid in generating supercharged extracellular vesicles

**Fig. 2:**
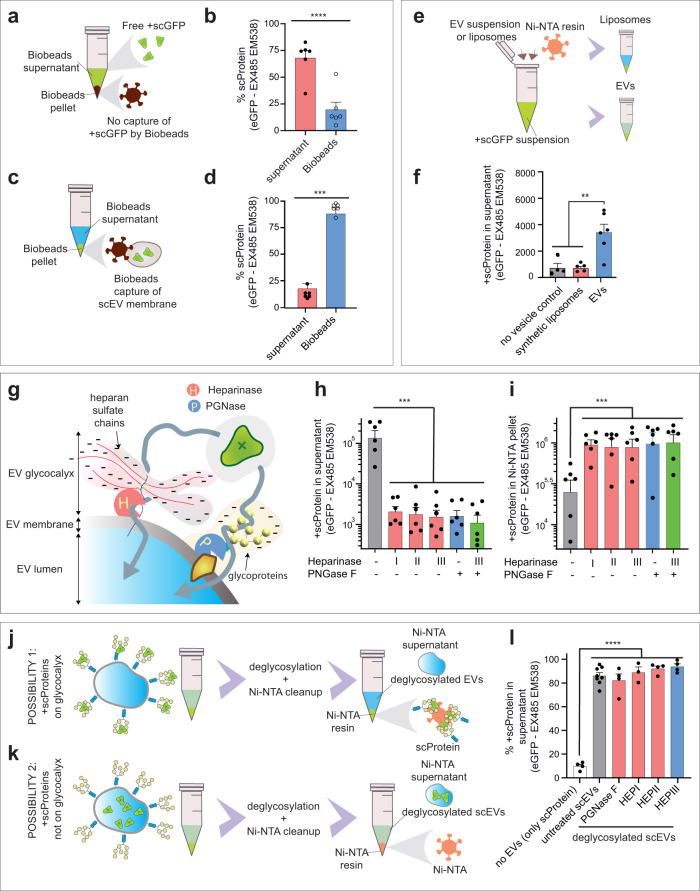
Membrane glycosylation is crucial for +scProteins to associate with extracellular vesicles (EVs). **a** Biobeads SM-2 are not able to pull-down +scProtein. Illustration that recombinant proteins, such as +scProteins, remain in the supernatant when incubated with Biobeads, which are known to interact with hydrophobic lipids. **b** Effect of Biobeads SM-2 incubation on recombinant +scProtein. Bar graphs represent the percentage of +scProtein fluorescence in Biobeads supernatant (*n* = 6) or Biobeads pellet (*n* = 6) after incubation with a +scProtein suspension without EVs. **c** Pull-down of scEVs with Bio-Beads SM-2. Illustration that scEV post-Ni-NTA incubation are pelleted by Biobeads due to their membrane content. **d** Effect of Biobeads SM-2 incubation on scEVs. Bar graphs represent the percentage of +scProtein fluorescence in Biobeads supernatant (*n* = 6) or pellet (*n* = 6) after incubation with scEVs. scEVs were generated with Ni-NTA cleanup and are derived from HEK293T cells. **e** Illustration of use of Ni-NTA resin to detect +scProtein and EV or liposome association. **f** +scProteins interact with EVs but not with liposomes. Bar graph represent the +scProtein fluorescence in the Ni-NTA supernatant in following conditions: +scProtein without EVs (no vesicle control, *n* = 4), +scProtein with negatively charged DPPC-PEG(2000)-DSPE-cholesterol liposomes (synthetic liposomes, *n* = 5), and +scProtein with HEK293T EVs (EVs, *n* = 6). **g** Graphical representation of the negatively charged glycosylated moieties on the surface of EVs as a potential +scProtein loading platform. Sulfated domains of heparan sulfate or oligosaccharides of glycoproteins can be removed by Heparinase [H] and PGNase [P], respectively. **h** EV loading with +scProtein is inhibited by enzymatic deglycosylation. HEK293T EVs (*n* per group = 6) were treated with PGNase F, Heparinase I, Heparinase II, or Heparinase III and exposed to Ni-NTA affinity resin. Bar graphs represent the +scProtein fluorescence in supernatant post-Ni-NTA pull-down. **i** scEV loading with +scProteins is EV glycosylation dependent. Bar graphs represent the +scProtein fluorescence in Ni-NTA pellet post-Ni-NTA pull-down shown in (**h**). **j** Tracking the position of +scProtein in scEVs with deglycosylation assay. Possibility 1 illustrates scEVs as a platform for +scProteins whereby the latter stick to the outer surface. Deglycosylation of scEVs in this possibility 1 results in +scProtein capture by Ni-NTA resin. **k** Tracking the position of +scProtein in scEVs with deglycosylation assay. Possibility 2 illustrates scEVs as a stable scProtein-EV assembly whereby deglycosylation does not result in Ni-NTA resin capture of +scProtein fluorescence. **l** Deglycosylation of scEVs does not disrupt +scProtein-EV assembly. Bar graph represents the percentage of +scProteins fluorescent signal in Ni-NTA supernatant vs Ni-NTA pellet in following conditions: only +scProtein (*n* = 4), untreated HEK293T scEVs (*n* = 8), and deglycosylated HEK293T scEVs (*n* = 4). These results confirm possibility 2 illustrated in (**k**). HEP = Heparinase. Data are presented as mean and SEM (error bars) and analyzed with unpaired t-test or one-way ANOVA. **, *** and **** represent a *p*-value of ≤ 0.01, <0.0001 to <0.001 and <0.0001, respectively.

Our results support the ability of positively charged residues of +scProtein to associate with BVs, such as EVs. Here, we investigated whether EV properties also aid in +scProtein association. Bio-Beads SM2 are nonpolar polystyrene adsorbents that are not expected to bind to hydrophilic +scGFP (Fig. 2a) but should be able to pellet phospholipids^21^ present on EVs. When these Bio-Beads were mixed with free +scGFP in solution 71.5 ± 15.2 of the fluorescence stayed in suspension (Fig. 2b). However, when scEVs were mixed with Bio-Beads SM2 (Fig. 2c), the fluorescence was reduced to 11.9 ± 13.5% in suspension confirming the capture and pelleting of +scGFP by cell-derived biomembranes present in EVs (Fig. 2d). We explored whether the lipids in EV membranes are the main determinant underlying association with +scProtein. Therefore, we compared +scProtein association with liposomes consisting of dipalmitoylphosphatidylcholine-polyethylene glycol 2000-distearoylphosphatidylethanolamine-cholesterol with a known surface charge of −11 ± 1 mV to that of EVs (Fig. 2e). After Ni-NTA pull-down, the +scProtein fluorescence in suspension with EVs was 6.29-fold higher compared to the liposome condition and 6.06-fold higher than the no vesicle control (scProtein alone) (Fig. 2f). We hypothesized that negative charges on the surface of BVs might be sugar moieties, which are lacking in liposomes. Therefore, EVs were treated with different enzymes, such as PGNase F or different heparinases (I, II, and III), to remove N-glycosylated moieties and thereby reduce the negative charge of their biological membranes^22^ (Fig. 2g). These enzyme treatments significantly decreased the +scProtein fluorescence in the supernatant after Ni-NTA pull-down, indicative of the level of +scProtein capture by EVs (Fig. 2h). Compared to non-treated samples a 74.1-, 69.13-, 84.70-, 92.34-, and 112.5-fold decrease in the supernatant was observed for a pretreatment with heparinases (I, II, and III), PGNase F and a mixture of Heparinase III with PGNase F, respectively. As a consequence of the decreased association of +scProtein by EVs, an increase in +scProtein fluorescence in the Ni-NTA pellet was observed for the deglycosylated EV conditions. Compared to non-treated samples a 3.16-, 3.68-, and 3.58-fold increase was observed for a pretreatment with heparinases (I, II, and III), PGNase F and a mixture of Heparinase III with PGNase F, respectively (Fig. 2i). Deglycosylation also enabled us to evaluate the stability of our established scEV complex. Here, we remove sugar moieties after +scProtein association with EVs, instead of our previously performed EV deglycosylation before +scProtein loading. If the sugar moieties on the extraluminal side merely mask the +scProtein interaction with the Ni-NTA, we expected that +scProtein on scEVs would detach after deglycosylation (Fig. 2j). However, if the association of +scProtein with scEVs was stable, a deglycosylation treatment would have no effect (Fig. 2k). No significant additional pull-down of Ni-NTA was observed after deglycosylation of scEVs with heparinases (I, II, and III) or PGNase F compared to untreated scEVs (Fig. 2l). These results substantiate that removing oligosaccharides after loading +scProtein does not influence the stability of the scEV assembly.

### Uptake of supercharged extracellular vesicles and supercharged virus-like particles in cultured cells

**Fig. 3:**
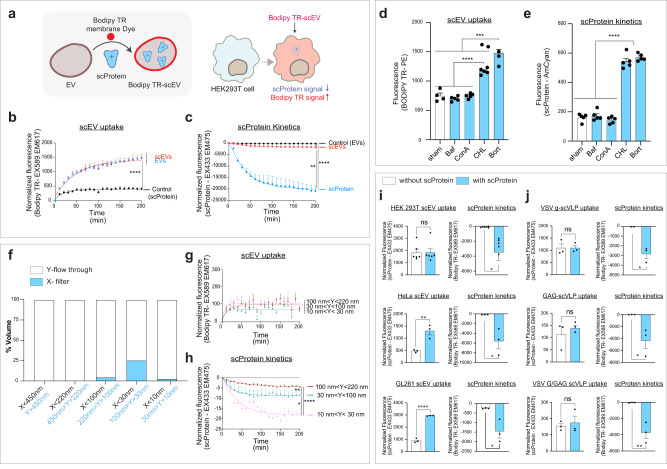
Monitoring uptake of supercharged extracellular vesicles (scEVs) and supercharged virus-like particles (scVLPs) with HEK293T recipient cells. **a** Monitoring EV uptake and scProtein kinetics with Bodipy TR-scEVs. Cartoon showing that the membrane of scEVs can be labelled with Bodipy TR Ceramide to study the uptake of scEVs by recipient cells. scProtein fluorescent signal can be monitored to study whether +scProtein is protected from cellular degradation when associated with EVs (exposure of scEVs vs scProtein without EVs). Bodipy TR signal increases with increased cellular take up, while scProtein fluorescence decreases with increased cellular degradation. **b** scEV uptake by cells. Bodipy TR fluorescent signal was monitored to measure difference in uptake between supercharged and non-supercharged EVs (exposure of scEVs vs EVs). HEK293T Bodipy TR-scEVs (scEVs, *n* = 3) and HEK293T BODIPY TR-EVs (EVs, *n* = 3) were incubated with HEK293T cells and Bodipy TR fluorescence was measured in cells. +scProtein (control, *n* = 3) exposure without EVs was monitored as a noise control for Bodipy TR fluorescence. Fluorescence measurements were performed every 10 min for 200 min. Curves indicate are nonlinear regression models for scEVs (R2 = 0.85) and EVs (R2 = 0.92). Each datapoint was normalized to its starting value. **c** scProtein kinetics post-scEV uptake. scProtein fluorescent signal was monitored to measure the cellular degradation between unassociated (free scProtein) and EV-associated scProtein (scEVs). HEK293T Bodipy TR-scEVs (scEVs, *n* = 3) and +scProtein without EVs (scProtein, *n* = 3) were incubated with HEK293T cells and scProtein fluorescence was measured over time. HEK293T Bodipy TR-EVs without scProtein (control, *n* = 3) was monitored as a noise control for +scProtein fluorescence. Fluorescence measurements were performed every 10 min for 200 min. Curves indicate are nonlinear regression models for scEVs (R2 = 0.92) and +scProtein (R2 = 0.72). Each datapoint was normalized to its starting value. **d** Effect of inhibitors on Bodipy TR-scEVs uptake by cells. HEK293T cells were pretreated with DMSO (sham, *n* = 5), v-ATPase blockers – 200 nM Bafilomycin A1 (Baf, *n* = 5) and 200 nM Concanamycin A (ConA, *n* = 5), 10 μM lysosomotropic Chloroquine (CHL, *n* = 5), or the 26 S proteasome inhibitor 5 μM Bortezomib (Bort, *n* = 5) 1 h before incubation with HEK293T Bodipy TR-scEVs. After 24 h of scEV exposure, Bodipy TR fluorescence in the recipient cells was acquired with flow cytometry. **e** Blocking scProtein kinetics with inhibitors. Effect of inhibitors on scProtein degradation by cells post-Bodipy TR-scEVs uptake. Conditions are explained in (**d**). scProtein fluorescence in the recipient cells was acquired with flow cytometry after 24 h of scEV exposure. **f** Generating scEVs with a different particle size. Separation of Bodipy TR-scEVs based on particle size by serial filtration with different cut-off filters. Each bar was normalized to 100% and represents the volume in the flow through (white) and the volume remaining in the filter (blue). **g** Particle size does not influence scEV uptake. HEK293T cells were incubated with different sizes of HEK293T Bodipy TR-scEVs. For each particle size scEV subpopulation (*n* = 3) Bodipy TR fluorescence was imaged every 10 min for 24 h. Each datapoint was normalized to the starting value and the residue volume. Curves indicate nonlinear regression models for 100 nm < Y < 220 nm (R2 = 0.75), 30 nm < Y < 100 nm (R2 = 0.30), and 10 nm < Y < 30 nm (R2 = 0.73). **h** Particle size of scEVs affect stability of scProtein in the recipient cell. +scProtein fluorescence post-scEV uptake by HEK293T cells was measured in conditions explained in (**g**). Curves indicate nonlinear regression models for scProtein fluorescence for 100 nm < Y < 220 nm (R2 = 0.24), 30 nm <Y < 100 nm (R2 = 0.12), and 10 nm < Y < 30 nm (R2 = 0.21). **i** Effect of EV origin on scEV kinetics. Bodipy TR-scEVs and Bodipy TR-EVs derived from HEK293T (*n* = 3) [autologous EVs], Hela (*n* = 3) [heterologous EVs], and GL261(*n* = 3) [heterologous EVs] cells were incubated with HEK293T cells for 200 min (see also Fig. S4f). Bars represent Bodipy TR and scProtein fluorescence after 200 min to model scEV uptake and scProtein kinetics, respectively. **j**  Effect of supercharged Virus-like particles (scVLPs) uptake by recipient cells. VLPs were generated by expressing VSV G (*n* = 3), GAG (*n* = 3), or VSV G/GAG (*n* = 3) in HEK293T cells and labelled with Bodipy TR. scVLP uptake and scProtein fluorescence was measured as explained in I (see also Fig. S4h). Fluorescence in time measurements was normalized to *t* = 0. Data are presented as mean and SEM (error bars) and analyzed with unpaired t-test, Kruskal–Wallis test or one-way ANOVA test. ****, ***, ** and * represent a *p*-value of <0.0001, <0.001, <0.01, and <0.05, respectively. R2 represents the statistical measure of how close the data are to the fitted regression line.

Uptake of EVs by recipient cells can be monitored with membrane dye labelled EVs^23^. To explore the uptake of scEVs by recipient cells, we fluorescently labelled scEVs with a Bodipy TR ceramide membrane dye^24^ (Fig. 3a). To generate our Bodipy TR-scEVs, EV samples were incubated with dye prior to supercharging EVs (Fig. S4a). Our method included resolving a Bodipy TR labelled EV suspension over a SEC column removing free dye visible in the higher non-EV SEC fractions (Fig. S4b-I) and a 100 kDa/~10 nm cut-off filter treatment to clean up any potential free +scProtein remaining in our suspension. Free +scProtein was demonstrated to pass through a 100 kDa cut-off filter but could be retained by a 30 kDa/~3 nm cut-off filter (Fig. S4b-II). Of note, non-supercharged Bodipy TR-EV controls were generated from the same EV source sample as our Bodipy TR-scEVs avoiding batch-to-batch variations between EV production (Fig. S4a). In our final suspension, the BODIPY TR-label co-immunoprecipitated with CD63 affinity beads, known to bind CD63^+^EVs, as did the +scProtein fluorescent signal (Fig. S4c).

When exposing HEK293T Bodipy TR-scEVs to HEK293T cells cultured in EV-free media, an increase in Bodipy TR cell-associated fluorescence was observed over a 200 min (1 measurement/10 min) monitoring experiment (Fig. 3b). The uptake of the non-supercharged Bodipy TR-EV controls was not significantly different than their Bodipy TR-scEV counterparts. Half of the Bodipy TR signal was reached after 170.8–227.5 min (R2 = 0.90) and 222.9–433.3 (R2 = 0.75) min for EVs and scEVs, respectively. Concomitantly with Bodipy TR fluorescence, we monitored +scProtein fluorescence as a measure of the half-life of the recombinant +scProtein (Fig. 3c). A decrease in +scProtein fluorescence was significantly greater (*p* < 0.01) when the recombinant protein was exposed to HEK293T cells without EV-association than when it was delivered in the scEVs format. The half-life of +scProtein without association with EVs was 25.4–42.00 min (R2 = 0.53), in contrast to 65.9–105.8 min (R2 = 0.73) with scEVs.

To examine the potential mechanisms of HEK293T scEV uptake, we pretreated HEK293T cells with inhibitors targeting the endolysosomal and autophagic pathways before adding Bodipy TR-scEVs. v-ATPase inhibitors concanamycin A (ConA, 200 nM) and Bafilomycin A1 (Baf, 200 nM) were used to investigate whether scEVs act through a potential pH-dependent uptake mechanism. Inhibitor pretreated HEK293T cells (1 h) were investigated 24 h post-exposure to Bodipy TR-scEVs with flow cytometry. No significant decrease in Bodipy TR was observed compared to sham-pretreated cells (Fig. 3d). However, pretreatment with a lysosomotropic agent, such as Chloroquine (CHL, 10 µM)^25^ for 1 h did significantly increase Bodipy TR signal fluorescence (*p* < 0.0001), indicating scEV accumulation in the recipient cells. Bortezomib (Bort, 5 µM), a 26S proteasome inhibitor, which also induces autophagy^26^ and ER stress^27^, increased scEV uptake compared to sham (*p* < 0.001). These inhibitors have been interchangeably used to track both particle uptake as lysosomal protease activity in cells^28^. Therefore, +scProtein fluorescence levels were measured and demonstrated to increase only compared to sham when pretreated with CHL or Bort (*p* < 0.0001, Fig. 3e). To investigate whether +scProtein fluorescence in cells is solely increased with Bort or CHL pretreatment by an elevated scEV uptake, we combined both treatments (Fig. S4d). Post-Bodipy TR-scEV uptake, Bodipy TR signal was not affected by the combination pretreatment (Fig. S4d-I), while +scProtein fluorescence in cells increased when treated with both Bort and CHL compared to CHL alone (Fig. S4d-II, *p* < 0.05). These observations suggest that +scProtein kinetics in a scEV recipient cell are not only dependent of scEV uptake and can be enhanced after scEV uptake by blocking protease activity in recipient cells with Bort. Of note, the cytoplasmic proteasome inhibitor Bort also increased +scProtein fluorescence in combination with Baf and ConA (*P* < 0.01).

HEK293T Bodipy TR-scEVs were separated based on particle size through serial filtration with 450, 220, 100, 30, and 10 nm cut-off filters to examine the effect of particle size (Fig. 3f). In contrast to free scProtein, which was only retrieved in a 3 nm cut-off filter (Fig. S4b-II), an scEV solution could be concentrated by 100, 30, and 10 nm cut-off filters thereby obtaining 100–220 nm, 30–100 nm, and 10–30 nm sized HEK293T Bodipy TR-scEVs, respectively. Based on Bodipy TR fluorescence, no significant difference in cell uptake was observed between different sized- Bodipy TR scEVs (Fig. 3g). In contrast, +scProtein half-life differed based on Bodipy TR-scEV particle size (Fig. 3h). In essence, larger (100–220 nm) scEVs had an intracellular half-life of 25.3–61.0 min (*p* < 0.01, R2 = 0.75) while the smaller (10–30 nm, and 30–100 nm) scEVs had a half-life of 7.1–43.6 min (*p* < 0.0001, R2 = 0.73). This data suggests that smaller scEVs which carry less +scProtein (Fig. 1m) are potentially more susceptible to cell endosomal degradation compared to larger scEVs with larger payloads.

In contrast to the use of homologous scEVs, Bodipy TR-scEVs from other cell sources were tested for their internalization potential on common recipient HEK293T cells. HeLa EVs and GL261 EVs had a similar Nanosight Analysis profile before being labelled with Bodipy-TR, supercharged with +scProtein, and exposed to HEK293T cells (Fig. S4e-I). Based on Bodipy-TR fluorescence, HeLa EVs (*p* > 0.05) and GL261 EVs (*p* < 0.05) did not well accumulate over a 200 min incubation at 37 °C with HEK293T cells compared to control (blue graph compared to black graph in Fig. S4f-I and II). However, when GL261 EVs (*p* < 0.0001) and HeLa EVs (*p* < 0.0001) were supercharged, their uptake by HEK293T cells was increased significantly compared to unsupercharged GL261 EVs and HeLa EVs (red graph compared to blue graphs in Fig. S4f-I, II). When comparing to homologous uptake of scEVs with heterologous scEV uptake by HEK293T cells, Bodipy TR fluorescence increases in the latter with supercharging (Fig. 3i-left bar graphs, Fig. S4f, g) but does not influence the +scProtein fluorescence in the cell (Fig. 3i-right bar graphs and Fig. S4g).

To verify whether in addition to the origin of EVs, viral factors could influence the uptake of supercharged BVs, virus-like particles (VLPs) were generated from HEK293T cells by overexpression of VSV G, GAG, or both VSV G and GAG (Fig. S4e-II). Similar to what we earlier observed with HEK293T EVs, no significant difference in uptake between Bodipy TR labelled supercharged and non-supercharged HEK293T VLPs was observed when incubated with HEK293T cells (left graphs in Fig. 3j and Fig. S4h). +scProtein fluorescent levels delivered with scVLPs did not differ between conditions in recipient HEK293T cells (right graphs in Fig. 3j and Fig. S4h).

Altogether, our data indicate that scEV uptake and scProtein kinetics in recipient cells can be influenced by particle size and EV source, but not by endogenous EV loading of viral components.

### Leakage of supercharged proteins from lysosomal compartments after cellular uptake of supercharged extracellular vesicles and supercharged virus-like particles

**Fig. 4:**
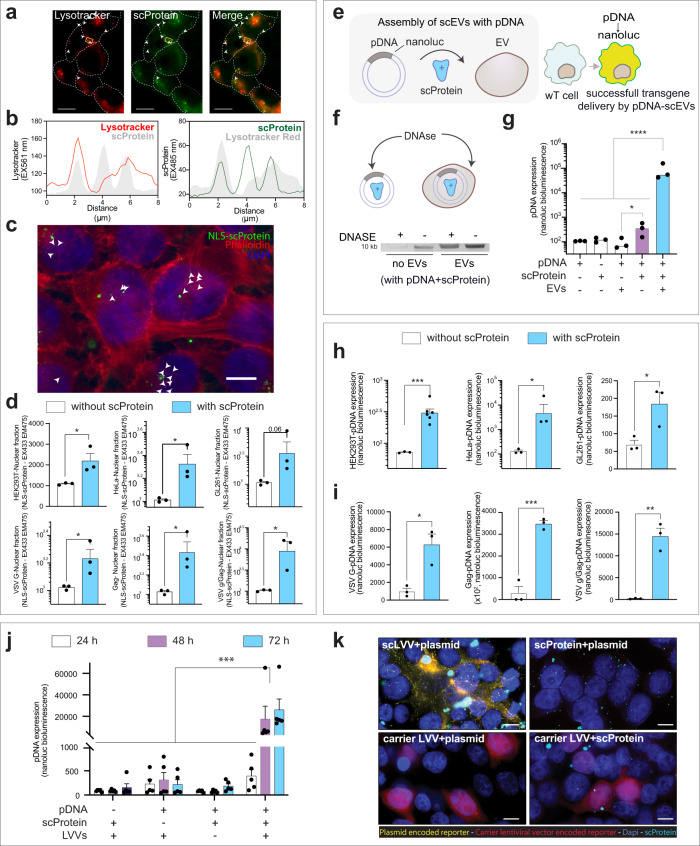
Downstream fate of +scProtein and plasmid DNA cargo post-supercharged biovesicles uptake. **a** scProtein delivered with scEVs leak from the endolysosomal compartments post-scEVs uptake. HEK293T scEVs were exposed for 3 days to HEK293T cells prior to endosomal compartment labelling with Lysotracker Red DND 990 (50 nM). White arrows highlight fluorescent +scProtein signal that is not overlapping with Lysotracker Red. Scalebar is 20 µm. **b** Lysosomal escape of +scProtein post-scEV uptake by cells. A line profile of Lysotracker Red (left) and +scProtein (right) fluorescent intensities observed in the yellow square in (**a**) were analyzed and presented here. +scProtein fluorescence graph displayed two peaks of fluorescence that overlap with Lysotracker Red graph and a third one (middle peak) that does not overlap with the Lysotracker Red signal. **c** Nuclear translocation of NLS-scProtein post-scEVs uptake. HEK293T EVs were loaded with a +scProtein coupled to a nuclear localization signal (NLS-scProtein). Fluorescent images of HEK293T cells exposed to NLS-scEVs for 3 days were screened for NLS-scProtein fluorescence (Fitc channel-cyan) in the cell nucleus. Actin filaments in recipient cells were stained with phalloidin (Cy3 channel-red). White arrows highlight fluorescent NLS-scProtein signal in nucleus (DAPI channel-blue), which does not colocalize with red. Scalebar is 10 µm. **d** Nuclear cell fraction isolation to quantify nuclear translocation of NLS-scProtein post-scEVs uptake. Cell nuclei were isolated from HEK293T cells which had been exposed for 3 days to different scEVs, including HEK293T scEVs, HeLa scEVs, GL261 scEVs, VSV G-scVLPs, GAG-scVLPs, and VSV G/Gag-scVLPs (*n* per condition=3). Bars represent fluorescent intensity of NLS-scProtein from isolated cell nuclei of HEK293T cells exposed to either BVs without scProtein (white) or supercharged BVs (blue). **e** Functional cargo delivery with scEVs. Plasmid DNA (pDNA) mediated delivery of reporter transgene Nanoluciferase (pDNA-Nanoluc) with pDNA-scEVs. Cartoon illustrating pDNA association with purified EVs through +scProtein to generate pDNA-scEVs. When pDNA-scEVs are taken up by recipient cells, successful delivery of pDNA generates a bioluminescent signal through its encoded Nanoluc transgene. **f** DNA is protected from DNAse when assembled into pDNA-scEVs. 1 µg pDNA was associated with pDNA-scEVs and used to assess pDNA protection against DNAse I degradation (15 min). DNAse I treated vs untreated pDNA-scProtein and pDNA-scEVs samples were loaded on an 0.75% agarose gel and visualized with GelRed Nucleic Acid dye. **g** pDNA expression in recipient cells mediated by pDNA-scEVs. pDNA-scEVs generated with 1 µg pDNA and HEK293T EVs were incubated with HEK293T cells for 4 days and compared to conditions lacking either one or two components of the pDNA-scEV assembly (i.e., pDNA, +scProtein, or EVs, *n* = 3 each). Bars represent the bioluminescence measured in the cell media of recipient cells resulting from transgene expression. **h** pDNA-scEVs mediated delivery with EVs derived from different donor cell species. pDNA-scEVs generated with 1 µg pDNA and HEK293T (*n* = 3), HeLa (*n* = 3), or GL261 (*n* = 3) EVs were incubated with HEK293T cells for 4 days. Bars represent the bioluminescence measured in the cell media of recipient cells resulting from pDNA expression among conditions with pDNA and EVs without +scProtein (white) or with +scProtein (blue). **i** pDNA mediated delivery with pDNA-supercharged virus-like particles (scVLPs). pDNA-scVLPs generated with 1 µg pDNA and VLPs derived from HEK293T cells expressing VSV G (*n* = 3), GAG-VLPs (*n* = 3), or VSV G/GAG-VLPs (*n* = 3) were incubated with HEK293T cells for 4 days. Bars represent the bioluminescence measured in the cell media of recipient cells resulting from pDNA expression between conditions with pDNA and BVs without +scProtein (white) or with +scProtein (blue). **j** Nanoluc transgene expression post-pDNA delivery mediated by supercharged lentiviral vectors (scLVVs) transporting a mCherry transgene. pDNA-scLVVs generated with 1 µg pDNA (Nanoluc-transgene) and HEK293T LVVs (mCherry-transgene) were incubated with HEK293T cells for 3 days and compared to conditions lacking one component of the pDNA-scLVV assembly (i.e., pDNA, +scProtein, or LVVs). pDNA-Nanoluc expression was highly increased when the 3 components of the pDNA-scLVV assembly were present. Bars represent the bioluminescence measured in the cell media of recipient cells resulting from pDNA expression (*n* = 5). Different bars represent different timepoints. **k** Fluorescent images of recipient pDNA-scLVV cells. 72 h post-pDNA-scLVVs exposure of HEK293T cells pDNA-Nanoluc transgene expression is shown in yellow (anti-FLAG), LVV transgene in red (mCherry), and +scProtein in cyan (scProtein). Scalebar represents 10 µm. Data presented with mean and SEM (error bars) and analyzed with unpaired t-test, or one-way ANOVA test. When needed, data were transformed to log10 to qualify for test assumptions. ****, ***, ** and * represent a *p*-value of <0.0001, <0.001, <0.01, and <0.05, respectively.

Our previous observations with inhibitors of endolysosomal function suggested this route of EV internalization by cells. Here, we marked the low pH compartments in a scEV-recipient HEKT293T cell with LysoTracker Red^29^ and investigated its position compared to +scProtein (Fig. 4a). Seventy-two h after scEV exposure, we determined that +scProtein was present in both Lysotracker positive and Lysotracker negative cell compartments (Fig. 4b). To confirm +scProtein leakage from endosomes post-scEV exposure, we loaded +scProteins equipped with a nuclear localization signal (NLS) into EVs. The resulting NLS-scEVs were incubated for 3 days with HEK293T cells and screened for +scProtein fluorescence in the nucleus (Fig. 4c). Isolated nuclei from recipient HEK293T cells after 4 days of NLS-scEVs or NLS-scVLPs exposure were then compared to nuclei of HEK293T cells exposed to EVs and VLPs from the same EV batch, but which had not been supercharged (Fig. 4d). A significant difference in +scProtein fluorescence of the isolated nuclei was observed for HEK293T scEVs (*p* < 0.05), HeLa scEVs (*p* < 0.05), VSV G-scVLPs (*p* < 0.05), GAG-scVLPs (*p* < 0.05), or VSV G/GAG-scVLPs (*p* < 0.05), while for GL261 scEVs we only noted a trend (*p* = 0.06) compared to non-supercharged EV or VLP controls. In line with +scProtein accumulation in cells between different EV sources (Fig. S4g-III), no differences were observed for nuclear translocation between HEK, HeLa, and GL261 scEVs. In contrast, loading multiple viral components such as VSV G and GAG increased nuclear translocation compared to GAG alone (*p* < 0.05). These results indicate again that scEV uptake by cells does not predict +scProtein kinetics.

### Supercharged extracellular vesicles, supercharged virus-like particles, and supercharged lentiviral vectors mediate plasmid DNA expression in recipient cells

It has been reported that plasmid DNA (pDNA) can be piggybacked into cells with scProteins^15^. We tested whether EVs associated with both +scProtein and pDNA would generate a pDNA encoded signal in the pDNA-scEVs recipient cells (Fig. 4e). The plasmid used for these experiments encoded a nanoluciferase reporter (see Supplementary Table 2) that enables bioluminescent recordings upon expression by cells. We tested the stability of the pDNA-scEV assembly using DNase I treatment which was able to degrade pDNA in the presence of EVs without +scProtein (Fig. S5). We observed that when pDNA is associated with +scProteins, the pDNA is still susceptible to DNase I treatment, but not when that combination is assembled with EVs as a pDNA-scEV complex (Fig. 4f). The HEK293T pDNA-scEVs assembly, containing all three components (EVs, scProtein, and pDNA), was incubated with HEK293T cells for 4 days (Fig. 4g). pDNA expression in the recipient cells was established by a detectable bioluminescence signal. This signal was significantly higher compared to other conditions whereby cells were exposed to only two components (+scProtein and pDNA or EVs and pDNA, *p* < 0.0001) or to single components (pDNA or EVs, *p* < 0.0001). As published in earlier reports^30^, the condition without EVs (pDNA and scProtein) was also able to significantly increase the bioluminescent signal compared to condition without scProtein (pDNA and EVs), but to a 4.5-fold lower extend than our pDNA-scEVs assembly (*p* < 0.0001). Moreover, we evaluated whether this pDNA-mediated delivery of HEK293T pDNA-scEVs was solely restricted to HEK293T EVs (Fig. 4h). Therefore, GL261, HeLa, or HEK293T derived EVs were used to generate pDNA-scEVs and control suspensions (pDNA and EVs without +scProtein). Based on bioluminescent readings, GL261 pDNA-scEVs (*p* > 0.05) and HeLa pDNA-scEVs (*p* > 0.05) were also able to induce pDNA expression in HEK293T cells after 4 days of incubation compared to the control suspensions without +scProtein but with pDNA and GL261 EVs or HeLa EVs, respectively. Similar conclusions to the NLS-scEV experiments, there was no difference in nanoluc expression observed between HEK293T, HeLa, or GL261 pDNA-scEVs observed. The same observation was made with HEK293T pDNA-scVLPs and HEK293T recipient cells (Fig. 4i). VSV G-scVLPs (*p* < 0.05), GAG-scVLPs (*p* < 0.001), or VSV G/GAG-scVLPs (*p* < 0.01) increased bioluminescence in HEK293T cells compared to control suspensions without +scProtein, but with pDNA and VSV G-VLPs, GAG-VLPs, or VSV G/GAG-VLPs, respectively. No significant differences were observed between the different scVLPs conditions in terms of nanoluc bioluminescence. We also investigated whether HEK293T LVVs (lentiviral Vectors) carrying a mCherry transgene could be complexed to +scProteins and pDNA generating pDNA-scLVVs. Different conditions consisting of combinations of LVV, pDNA, and +scProteins were exposed to HEK293T cells for 3 days (Fig. 4j). Only the pDNA-scLVV assembly with all components (scLVV = +scProtein, LVV, and pDNA) being present was able to express detectable levels of bioluminescence. The pDNA-scLVV induced signal was 119–169-fold higher as compared to controls. Data could be confirmed with immunohistochemistry detecting Flag-tag present in our pDNA encoded protein (yellow) in less than 0.01% of the pDNA-scLVV recipient cells, and absent in cells after control treatments (Fig. 4k). Overall, our data confirm that +scProteins assembled with EVs, VLPs, or LVV and pDNA mediate pDNA expression in recipient cells.

### Supercharged lentiviral vectors as a model for a future multi-cargo delivery modality in the brain

**Fig. 5:**
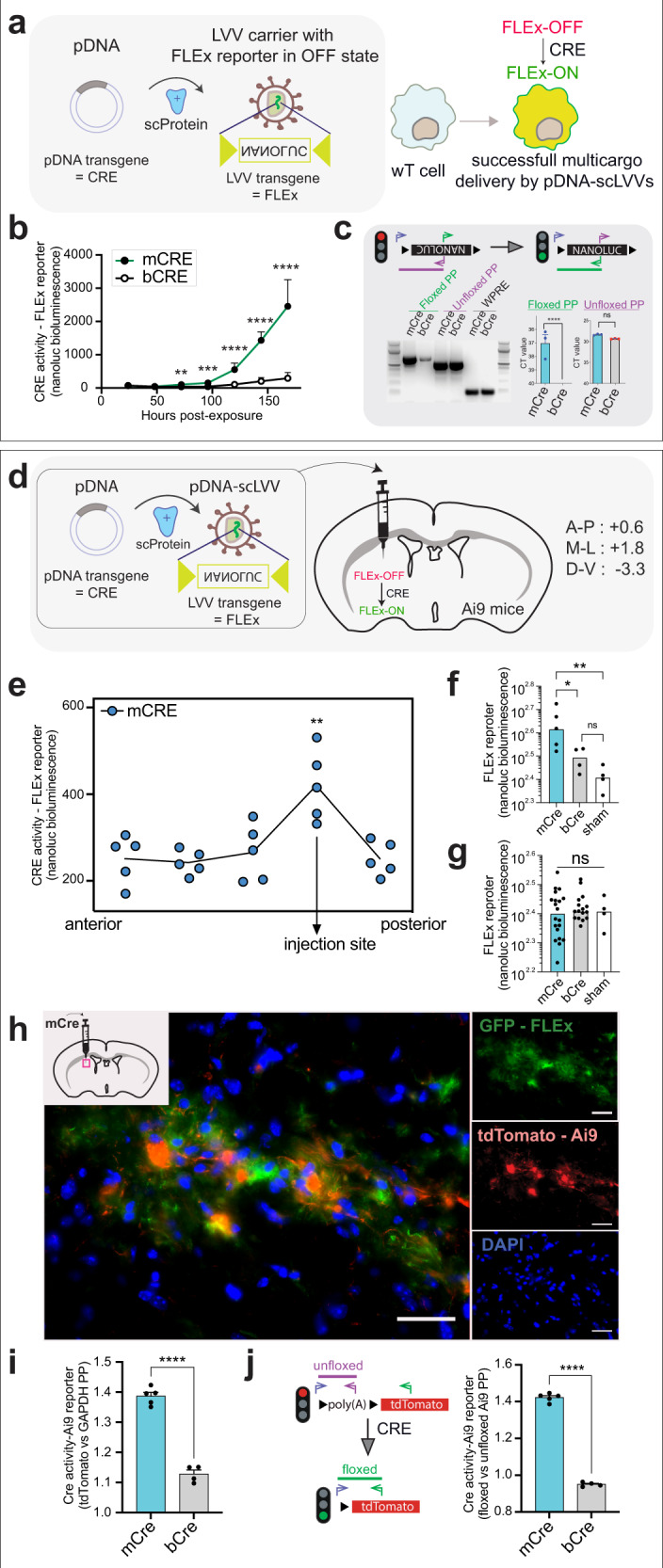
Two component delivery with supercharged lentivius vectors (scLVVs) to single cell demonstrated through bioluminescent reporter activity in vitro and in vivo. **a** FLEx reports on complex payload delivery with pDNA-scLVVs. Illustration describing that a plasmid (pDNA) encoding for CRE is associated with our supercharging procedure to a lentiviral vector (LVV) encoding for a FLEx reporter. The FLEx reporter encodes for an inverted version of Nanoluc DNA between the LoxP sites (FLEx-OFF). A bioluminescent signal in a recipient cell necessitates delivery of both CRE and FLEx transgenes by our pDNA-scLVV modality to a single cell. CRE acts on the FLEx (FLEx-OFF) reporter transgene to flip Nanoluc in frame with the promotor (FLEx-ON). **b** pDNA-scLVV delivers both Cre and FLEx transgenes to recipient cells. 1 μg of pDNA was loaded into pDNA-scLVVs whereby the CRE was preceded by the mammalian promoter EF1α (mCre, *n* = 4), which is active in mammalian cells or the bacterial active lac promoter (bCre, *n* = 4), which is inactive in mammalian cells. Graph represents bioluminescence readings over time after pDNA-scLVVs exposure to HEK293T cells. **c** FLEx activity post-pDNA-LVVs uptake by recipient cells confirmed with qPCR. Schematic representation showing FLEx reporter analysis on genomic DNA with primer pairs distinguishing between the floxed (green amplicon) and unfloxed (purple amplicon) state of the FLEx reporter (ON- vs OFF-state). Agarose gel illustrating the 615 bp floxed amplicon and the 534 bp unfloxed amplicon of the FLEx reporter following 7 days of pDNA-scLVVs exposure. Floxed and unfloxed FLEx amplicons were quantitatively compared with qPCR between cells exposed to pDNA(mCre)-scLVV (*n* = 3) or pDNA(bCre)-scLVV (*n* = 3). **d** Delivery of multi-component payload to the brain with pDNA-scLVVs. Cartoon describing intracranial injection of the pDNA-scLVVs platform containing pDNA-Cre and LLV-FLEx in Ai9 reporter mice. Successful delivery of pDNA-scLVVs encoded transgenes can be verified with FLEx and Ai9 reporter in striatal cells through activation of nanoluc and tdTomato expression, respectively. **e** Activation of pDNA-scLVV delivered FLEx reporter in the brain. 80 µm sections were taken for analysis from Ai9 mice that received pDNA(mCre)-scLVV ranging from anterior to posterior brain regions. Nanoluc bioluminescence as an indicator for FLEx activation was measured in each section (*n* = 5 per section). The peak of bioluminescence indicates the injection site. **f** FLEx reporter activity at pDNA-scLVV injection site. Brain sections at injection site were compared based on bioluminescence between mice exposed to pDNA(mCre)-scLVV (test, *n* = 5), pDNA(bCre)-scLVV (control, *n* = 4), or sham (*n* = 4). Significant differences were only observed at the injection site. **g** FLEx reporter activity outside pDNA-scLVV injection site. Brain sections at other regions outside injection site were compared similar to (**f**). **h** Mouse Ai9 reporter to demonstrate local delivery of pDNA-scLVV transgenes in striatum. pDNA-scLVV recipient cells were identified through GFP encoded by the FLEx reporter and verified for Cre activity through tdTomato fluorescence because of Ai9 reporter activation. GFP and tdTomato were detected with αGFP and αRFP, respectively. Scalebar is 50 µm. **i** tdTomato expression levels as quantitative measure of Ai9 reporter activity with pDNA-scLVVs in the brain. tdTomato mRNA levels were measured in brain sections at injection site from mice injected with either pDNA(mCre)-scLVV (*n* = 5) or pDNA(bCre)-scLVV (*n* = 4). Data were normalized against GAPDH. **j** Floxing events to detect Ai9 reporter mice post-pDNA-scLVVs mediated delivery of Cre. Primer sets discriminating between the floxed and unfloxed Ai9 reporter mRNA levels were used as indicated in the cartoon. Floxing events of the Ai9 reporter were quantified in brains sections at injection site of mice injected with pDNA(mCre)-scLVV (*n* = 5) or pDNA(bCre)-scLVV (*n* = 4). Bars represent a ratio between floxed vs unfloxed Ct-values. Data presented with mean and SEM (error bars) and analyzed with unpaired t-test, or one-way ANOVA test. ****, ***, ** and * represent a *p*-value of <0.0001, <0.001, <0.01, and <0.05, respectively.

Our previous findings indicate that pDNA-scLVVs deliver both LVV- and pDNA-encoded transgenes using mCherry and a Nanoluc reporter, respectively. Here, we tested whether both transgenes (pDNA-transgene and LVV-transgene) can successfully be delivered to the same recipient cell and collaboratively generate a bioluminescent signal (Fig. 5a). We developed a model with a LVV that encodes a nanoluciferase-reporter transgene here called FLEx. FLEx in our LVV is in the OFF-state and can only be activated to the ON-state when pDNA encoding for Cre recombinase is co-delivered. We tested whether FLEx activation is possible with pDNA-scLVVs, when LLVs are carrying the FLEx-OFF transgene. We compared in these experiments pDNA-scLVV loaded with a Cre plasmid preceded by either a mammalian promoter (mCre-test) or a bacterial promoter (bCre - control). As expected, only the pDNA-scLVV with the mCre could induce FLEx-reporter activity in our recipient HEK-293T cells (*p* < 0.0001, Fig. 5b). We confirmed activation of the FLEx reporter in the mCre condition, absent in the bCre condition, with qPCR utilizing primer pairs discriminating between the ON- and OFF-state of FLEx at the gDNA-level in the recipient cells (Fig. 5c). Of note, under both pDNA-scLVV conditions (mCre and bCre) similar amount of LVV transgene integration (*p* > 0.05) could be detected in the genome of the recipient cells as assessed by qPCR for WPRE.

We revisited our FLEx model to test whether pDNA-scLVV can co-deliver two payloads (pDNA transgene and LVV transgene) to brain cells (Fig. 5d). mCre- or bCre- encoding pDNA-scLVVs suspensions were injected into the striatum of adult Ai9 reporter mice^31^, in which Cre can turn on tdTomato expression in mouse cells. Twenty-five days after injecting pDNA-scLVVs, Ai9 mice were sacrificed and checked for Cre and FLEx deployment in the brain. Sections (80 µm) were taken from different areas in the Ai9 brains to verify FLEx-reporter delivery and FLEx-activation as a result of Cre activity. We confirmed FLEx activity in the section at the injection site as the nanoluciferase signal was significantly higher (Fig. 5e, *p* > 0.01) compared to sections in anterior or posterior brain regions. Furthermore, Ai9 animals receiving bCre or mCre pDNA-scLVVs were compared at the injection site (Fig. 5f) and more distal brain regions (Fig. 5g). FLEx-activity was significantly higher for mCre as compared to bCre or sham injected animals at the injection site (*p* > 0.05), while no difference was observed in nanoluciferase signal in other sections among the conditions (*p* > 0.05). To confirm that Cre delivered by pDNA-scLVVs was not only able to flip our FLEx-switch, we also screened for tdTomato signal that results from activation of the mouse-encoded Ai9 reporter. pDNA-scLVV delivery to brain cells was identified at the injection site through GFP fluorescence encoded by the FLEx-transgene (Fig. 5h). In these cells, Ai9 tdTomato fluorescence was visualized with immunohistochemistry. We quantified the tdTomato transcript levels at the injection site in pDNA-scLVV injected animals and thereby showed that the Ai9 reporter was significantly activated by mCre, and not by bCre (*p* > 0.0001, Fig. 5i). We confirmed that the tdTomato signal resulted from the removal of a floxed region in Ai9 mice by Cre activity (Fig. 5j). Indeed, qPCR with discriminating primers between floxed and unfloxed regions confirmed floxing by Cre in Ai9 brain sections with mCre pDNA-scLVV compared to bCre pDNA-scLVV (*p* > 0.0001). As exemplified with pDNA-scLVVs, our data suggest that the delivery capacity of vesicles can be augmented through supercharging to deliver multiple types of biomolecules with a single carrier platform that work in a combinatorial way on recipient cells.

## Discussion

Delivery of cargo with scProteins to cells has proven to be feasible but can be improved by adding a lipid component^32^. Entrapment of negatively charged scProteins (−scProteins) within cationic lipids improves scProtein translocation into the cytosol and protection of their accompanied cargo against potential degradation^33^. In our setup, we provided a lipid component to +scProteins by using EV, VLP, and LVV membranes to achieve combinatorial functional delivery of cargo to recipient cells. Larger EVs were more prone to +scProtein loading compared to smaller ones, indicating that more luminal volume and interaction surface promote incorporation of +scProteins. The surface of EVs consists of negatively charged lipids, such as ceramides^24^ and phosphatidylserine^34^ restricting association with negative supercharged scProteins (−scProteins) and non-supercharged proteins (scaffold). This ensures that larger membrane-encompassed vesicles exert a higher negative charge (from −12.3.0 to −16.0 mV), compared to smaller EVs (−9.0 to −12.3 mV) and non-membranous exomeres (−2.7 to −9.7 mV)^35^. As an important factor, we noticed that our +scProtein did not associate with −11 mV synthetic liposomes in contrast to SEC-purified EVs. This observation is in line with earlier reports that the charge of lipids is less important for docking of Arg-rich peptides in contrast with sugar moieties on biomembranes^36^. It is known that the EV surface contains heavily glycosylated proteins that influences their uptake by cells^37^. EVs adopt sugars from their originating both plasma membrane and endocytic cell membrane compartments^38^. In this regard, the extracellular domain of tetraspanins are equipped with N-linked glycosylation sites^39^ important for endocytic membrane trafficking^40,41^. We’ve demonstrated that deglycosylated EVs were not able to integrate +scProteins and a high number of tetraspanins on the EV surface is propitious for +scProtein association. In terms of the % EVs loaded in a SEC purified EV sample, we expect that our supercharging method could be improved by +scProtein loading of EV subpopulations rich in tetraspanins such as CD63^+^CD81^+^EVs, with large particle size, with a high glycosylation status, and with a negative surface charge.

Supercharging of HEK293T EVs did not influence uptake but did increase +scProtein half-life following uptake by HEK293T cells. Improving scEV uptake through using scEVs from different sources did not increase +scProtein levels when exposed to the same cell type, indicating the scProtein half-life is dependent on process dictated by the recipient cell. Through lysotracker red experiments scEVs were found to be taken up into low pH cell compartments, indicating that scEVs are exposed to endolysosomal conditions. We hypothesize that the stability of scEV assembly inside cells aids in the scEV-mediated delivery of pDNA to a recipient cell. It is known that supercharging of proteins protects them against proteolysis and other physical stresses, such as denaturation by temperature or denaturation and aggregation by chemicals like 2,2,2-trifluoroethanol^15,30^. The resilience to many hazardous factors and tolerance of scProteins^42^ compared to amphipathic cell penetrating peptides^43^ makes them ideal in delivery of associated cargo to a living cell or organism. We’ve demonstrated that assembly formation through supercharging of EVs protects them against deglycosylation, DNAse activity, and degradation in recipient cells. Delivery of scEV assemblies containing a higher level of +scProteins, such as large scEVs (220–100 nm) compared to smaller scEVs (<100 nm) was accompanied with an increased half-life of cargo. Longer stability implies longer interaction with the endosomal compartments, therefore boosting the +scProteins ability to escape from endosomal compartments^44,45^. We confirmed nuclear translocation of the +scProtein, as well as delayed cytosolic +scProtein degradation, using the 26S proteasome inhibitor Bort in combination with CHL, BafA, and ConA. More importantly, pDNA delivery and expression were increased with pDNA-scEVs compared to +scProtein and pDNA alone.

Formation of pDNA-scEVs is built upon the potential of +scProteins to associate with genetic material mainly through their lysine residues^46^, while at the same time, protecting the associated DNA from degradation^15^. We utilized this +scProtein property to piggyback pDNA-scProtein complexes for entrance into EVs. Nanoluc expression in HEK293T cells confirmed transgene delivery by pDNA-scEVs through the detection of bioluminescence. We adapted this readout to corroborate whether supercharging of BVs might be applicable for delivery of multiple types of biomolecules through scLVVs. pDNA-scLVVs generated a nanoluc signal whereby two components CRE and a FLEx-OFF reporter were delivered to the same cell generating bioluminescent readout. The LVV component provided viral RNA encoding a non-active floxed reporter, while the pDNA component encoded a CRE enzyme able to activate the floxed reporter. Apart from nanoluc activity, secondary downstream analysis with qPCR confirmed FLEx reporter editing by Cre. This functional delivery of multiple biomolecule types by means of a single carrier was not only shown in cell culture but substantiated by activation of the Ai9 reporter in mouse brain cells.

Nanoscopic vesicles derived from cells in culture provide a valuable route for supercharging to enhance cargo loading, cellular uptake, and functional delivery of cargo (Box 1). Delivery of multiple types of biomolecules^47^ is a highly valuable tool for next-generation research and modern medicine^9^. With our supercharging method, we were able to overcome some of the hurdles seen with packaging EVs through natural and transgenic routes with donor cells, including payload inconsistency, reduced options for multicargo transport, limited control over the loading process, and the need for oversaturating recipient cells with loaded EVs to achieve functional responses^48^.

Box 1 Our study introduces a customizable BV-loading technique that is

- a two-step procedure (approx. 1 h),
- easily scalable (for in vitro and in vivo purposes),
- occurs under physiological conditions (no need for compromising the vesicle integrity or introducing non-physiologic pH or osmotic changes),
- very efficient (focuses on intact membrane vesicles and not on free protein contaminants),
- applicable to all natural glycosylated enclosed nanobiomembranes (EVs, LVVs, or VLPs),
- applicable to every BV source (no need for transgenic EV-donor cells),
- delivery efficacy of pDNA is tunable by varying vesicle properties,
- serves as combinatorial carrier of different types of biomolecules either as LVV transgene or pDNA that is functional in the recipient cell.

## Materials and methods

Detailed methods are provided in the Supplementary Materials and Methods of this paper.

### Cell culture

Human embryonic kidney 293 (HEK293T), GL261 cells, and HeLa cells were obtained from the American Type Culture Collection and were cultured at 37 °C in a 5% CO2 humidified incubator. Culture media was comprised of Dulbecco’s modified essential medium (DMEM) with L-glutamine (Corning) supplemented with penicillin (100 units/ml), streptomycin (100 mg/ml) (P/S) (Corning), and 10% fetal bovine serum (FBS) (Gemini Bioproducts). Stock cells were passaged 2–3 times/week with 1:4 split ratio and used within 8 passages. Cells were monthly tested for mycoplasma contamination (Mycoplasma PCR Detection Kit, abm G238) and found negative. Cells grown for EV isolation were cultured in media supplemented with 5% EV-depleted FBS (FBS was depleted of EVs by 16 h centrifugation at 160,000 × *g*).

### EV and biovesicle isolation from cells with size exclusion chromatography (qEV) column

EVs isolated from thirty ml of conditioned medium were collected from cells cultured at 70% confluency in two 100 mm plates after 72 h (seeding density 2.2 × 10^6^ cells/plate). The conditioned media was centrifuged at 300 × *g* for 10 min to remove intact cells, dead cells, and cell debris. The medium was then concentrated using a centrifugal concentrator with a 100,000 molecular-weight cutoff (Amicon^®^Ultra-15 Centrifugal filters), yielding about 0.5 ml concentrate (two spins of 10 ml at 6000 × *g* for 10 min). This concentrate was resolved by passing through IZON qEV original size exclusion columns (SEC) followed by 15 ml of double filtered (0.2 μm) PBS. Five-hundred-microliter fractions were collected. High particle/low protein fractions (from 7 to 11) were pooled and concentrated using Amicon^®^Ultra-0.5 Centrifugal filters to a final volume of 200 µL at 10,000 × *g* for 30 min. The typical yield of an EV isolation was approximately 7.1 × 10^7^ ± 3.2 × 10^7^ particles/ml. This method was adapted to isolate EVs, LVVs (transgene plasmid, psPAX2 (Addgene #12260) and pMD2.G (#12259), VSV G-VLPs (pMD2.G (Addgene #12259), and GAG-VLPs (psPAX2 (Addgene #12260)) before being exposed to scProteins (see below). LVVs purified from media of 2.5 million HEK293Tcells transfected with psPAX2 (Addgene #12260) and pMD2.G (Addgene #12259) were isolated with SEC.

### Loading of biovesicles with scProtein

5 × 10^12^ concentrated EVs (based on Nanosight measurement) were loaded in 50 µl with 283 nM recombinant scProtein and incubated for 15–45 min with gentle agitation on a HulaMixer™at room temperature. Then 50 µl Ni-NTA agarose resin (Qiagen) or NEBExpress Ni-NTA Magnetic Beads (NEB) in PBS were added, incubated for 15–30 min to 24 h on a HulaMixer™ and compared based on fluorescence to a control of scProtein and agarose resin (Qiagen) without EV suspension. After centrifugation for 30 s at 14,000 × *g*, the supernatant was collected leaving the resin with the bound scProtein that was not associated with the EVs. Fluorescence in suspension was visually inspected using a UV lamp with black background and quantified with a microplate reader (Synergy H1 Hybrid Multi-Mode Reader, BioTek) at an excitation wavelength of 485 nm for GFP or 433 nm for mCerulean3. Similarly, +scProtein solution was exposed to liposomes (100–200 nm vesicles based on Nanosight) at a concentration of 6.29 × 10^8^ particles/ml that were kindly provided by Dr. Van Solinge. The liposomes were diluted to match our EV sample with 7.5 × 10^7^ ± 1.2 × 10^7^ particles/ml (based on Nanosight). The liposomes used were negatively charged DPPC-PEG(2000)-DSPE-cholesterol liposomes, which have been characterized in depth by Deshantri et al. 2019^49^. This method was also adapted for loading LVVs, GAG-VLPs, VSV G-VLPs GAG/VSV G-VLPs. The carrier LVV was concentrated with spin filters (see above) and 30 µl of this suspension was loaded with both plasmid (1 µg) and scProtein (2.2 mol). The total solution of 90 µl was incubated with gentle agitation on a HulaMixer™ at 4 °C overnight to generate scLVV. Then 90 µl samples were added to cells at a density of 50,000 cells in each well of a 12-well plate. Genomic viral RNA (vRNA)-carrying EVs, GAG-VLPs and VSV G-VLPs were generated similarly to scLVV, but were generated either from cells expressing psPAX2 encoding pol and GAG (GAG-VLPs), or pMD2.G encoding for VSV G (VSV G-VLPs).

### scEV characterization with exoview

A sample of EVs purified from HEK293T cells (see above) was concentrated with spin filter columns (Milipore 30 kDa) to a final volume of 30 µl. These EVs were either loaded with 20 µl 283 nM scProtein (see procedure above) or remained unloaded at 4 °C overnight. According to the guidelines provided by NanoView Biosciences (USA), the samples were incubated on the ExoView Tetraspanin Chip for 16 h at room temperature. After washing the chips three times in 1 ml PBS for 3 min, they were incubated with ExoView Tetraspanin labelling antibodies (1:500 in PBST) with 2% BSA for 2 h. The chips were rinsed with PBS and then imaged with the ExoView R100 reader. Procedure and initial analysis were performed by the ExoView representative.

### scProtein production

One Shot^®^BL21 Star™ (DE3; Invitrogen) bacteria were transformed with plasmids encoding the scProtein of interest and plated on LB agar plates with appropriate antibiotic (kanamycin 50 µg/mL, Sigma). After overnight incubation at 37 °C, a medium sized, isolated colony was picked and inoculated into a 5 mL overnight seed culture of LB media. The seed culture was diluted 1:20 and grown in 2xYT media (Sigma) supplemented with 0.2 um filtered 40 mM MgSO4 (Sigma), 2.5 mM KCl (Sigma), and 20 mM glucose (Sigma) until obtaining OD600 ~0.1–0.3 and then, for protein expression, the bacteria were induced by adding 0.5 mM isopropyl β-d-1-thiogalactopyranoside (IPTG; Sigma) and incubated overnight at 37 °C. Cultures were harvested by centrifugation at 3000 × *g* for 10 min and pellets stored at −80 °C or processed immediately.

### Bio-beads pull-down of scEVs

Bio-Beads SM-2 resin (Biorad) were suspended in PBS to 0.2 g/ml. Subsequently, 50 μl of the Bio-Bead solution was added to a 500 μl scEV suspension generated from a mixture of 7.5 × 10^7^ +/− 1.2 × 10^7^ EV particles/ml and 10 pmol of +scProtein or a scProtein solution without EVs. The samples were incubated overnight at 4 °C degrees with gentle agitation on a HulaMixer™. As a control for Bio-Bead pull-down, a 10% Triton X-100 solution was incubated under the same conditions. The following day, the Bio-Beads were spun down (6000 × *g* for 1 min) and resuspended in 50 μl PBS. 50 μl of the supernatant and the Bio-Bead solution were measured with a Synergy H1 Hybrid Multi-Mode Reader (Biotek).

### Removal of N-glycosylated moieties

7.5 × 10^6^ EVs in 100 μl solution prior or after scProtein loading were incubated with 10 μl Heparinase I/II/III seperately from *Flavobacterium heparinum* (Sigma) or with PNGase F (New England Biolabs). For optimal enzyme activity, a buffer solution was added provided by the supplier and the samples were incubated for 24 h at 37 °C or 25 °C, respectively, in a HulaMixer Sample Mixer (Invitrogen). Ni-NTA affinity resin was added and pelleted (16,000 × *g* for 5 min) to exclude unloaded scProtein. scProtein fluorescence was measured after incubation for 15–30 min to 24 h in Ni-NTA supernatant and Ni-NTA resin pellet.

### CD63 immunoaffinity beads for scEV characterization

20 μl of antiCD63 beads (invitrogen) were washed twice with 0.1% BSA inPBS and provided with 100 μl suspensions of HEK293T scEVs, HEK293T EVs, scProteins, HEK293T Bodipy TR labelled scEVs, or HEK293T EVs. The samples were incubated overnight at 4 °C with gentle agitation on a HulaMixer™. Beads were washed twice with 0.1%BSA/PBS and when needed incubated with antiCD63-APC (MEM-259, Invitrogen) in a 100 μl volume with 0.1% BSA/PBS for 1 h at 4 °C with gentle agitation. Samples were washed twice by centrifugation (300 × *g* for 15 min) and dissolved in 250 μl 0.1%BSA/PBS for flow cytometry measurement.

### Labelling of scEVs and uptake

1.8–2.0 × 10^7^ EVs from HEK293T, HeLa, or GL261 cells in 30 μl PBS each were labelled with 10 µM Bodipy™ TR-ceramide (ThermoFisher) for 1 h on a HulaMixer at 37 °C (Fig. S4-A). To remove excess dye, EVs were isolated by SEC. Samples were divided such that one part was supercharged with 50 pmol scProtein, as described above, while the other part was incubated in the same volume of PBS. Lastly, the samples were applied to a 10 nm (~100 kDa) cut-off filter (milipore) as an additional step to eliminate free scProtein (30.1 kDa = ~3 nm). To monitor EV and scEV uptake, HEK293T cells (10,000 cells per well) were seeded in 96-well plates (Falcon) in optimem culture medium (Gibco) overnight in serum-free media. Images were acquired every 10 min for 200 min using a Synergy H1 Hybrid Multi-Mode Reader (BioTek). The average fluorescent intensity of scProtein and Bodipy TR was normalized to the starting value. Flow cytometry experiments of scEV uptake after 24 h were performed, as described below, separately from 200 min monitoring experiments.

For inhibitor treatment before scEV uptake, HEK293T cells (50,000 cells per well) were seeded in 24-well culture plates (Falcon) for 24 h in DMEM with serum. The cells were then pre-incubated with the inhibitors in Optimem without serum. The following inhibitors were used: 200 nM bafilomycin A1 (Sigma-Aldrich), 200 nM concanamycin A (Sigma-Aldrich), 100 µM Chloroquine (Sigma-Aldrich) or 5 µM Bortezomib (Millipore). The labelled EVs (10^7^–10^8^ particles) were added each well for 24 h in the presence of the indicated inhibitors. Following incubation, cells were trypsinized and analyzed based on scProtein and Bodipy TR fluorescence on a Beckman SORP 5 Laser BD Fortessa Flow Cytometer in the MGH core facility.

For uptake of different EV sizes, 3 × 10^8^ per 0.5 ml BODIPY-TR labelled scEVs were serial filtered through 450 nm (Costar), 220 nm (Costar), 100 nm (Costar), 300 kDa (~30 nm, Millipore), 100 kDa (~10 nm, Millipore), and 30 kDa (~3 nm, Millipore) cut-off filters. Images were acquired every 10 min for 200 min using a Synergy H1 Hybrid Multi-Mode Reader (BioTek). The average fluorescent intensity of scProtein and Bodipy TR was normalized to the starting value.

### Nucleus isolation post-scEV exposure of hek293t cells

10^7^–10^8^ scEVs and EVs derived from HEK293T, HeLa, and GL261 cells were incubated with 50,000 HEK293T cells per 24-well. After 4 days of incubation, cells were trypsinized and washed with PBS. Cell fractionation kit (Abcam,109718) was used to extract cytosolic, mitochondrial, and nuclear proteins. scProtein fluorescence was measured with a Synergy H1 Hybrid Multi-Mode Reader (BioTek).

### Assembly of scEVs with plasmid DNA (pDNA)

10^7^–10^8^ HEK293T EVs, GL261 EVs, HeLa EVs, VSV G-VLPs, GAG-VLPs, VSV G/GAG-VLPs, and LVVs were incubated with 50–100 pmol scProtein and 1 μg pDNA overnight at 4 °C. Binding of pDNA to complexes was verified on 1% agarose gels. To test the stability of assembly, 2 units of TURBO Dnase I (Invitrogen) was added at 37 °C for 15 min and compared to pDNA/scProtein or pDNA with and without DNase treatment. pDNA used for this experiment has been summarized in Supplementary Table 1 and published in the Rufino-Ramos et al. 2022 manuscript^50^.

### Bioluminescent and fluorescent assays

Recipient cells were trypsinized and seeded in 24-well plates (50,000 cells/well) in 500 μl complete DMEM media. After 24 h, 100 μl of the pDNA-scEV suspension was added to the cultured cells. Each day nanoluciferase^37^ was monitored by removing 50–100 μl of media. Nanoluciferase expression was analyzed with the addition of furimazine (Nano-Glo^®^Luciferase, Promega) diluted in 1× PBS in a range from 1:250 to 1:500. Samples were incubated with the reagent for at least 3 min prior to reading on Synergy H1 Hybrid Multi-Mode Reader (BioTek). For luminescent readings, samples were loaded into white 96-well culture plates (Lumitrac 200). For fluorescent readings, the samples were loaded into black 96-well culture plates (10,000 cells/well). Each sample was loaded in triplicate with a volume of 100 µl in each well. Biovesicles were loaded with pDNA as listed above. Coronal tissue samples from mouse brain corresponding to 150 μm thick sections were homogeneized in 500 μl Nano-Glo^®^ Luciferase Assay Buffer. Bioluminescence were analyzed by adding 100 μl sample and 100 μl 1:250 furimazine (Nano-Glo^®^ Luciferase, Promega) in 1xPBS. The excitation laser was shut off, and the emitted light was measured at two different gains: 135 and 200.

### Animals

All animal experiments were conducted under the oversight of the MGB Institution Animal Care and Use Committee. Ai9 mice^31^ were maintained with unlimited access to water and food under a 12 h light/dark cycle. Male and female Ai9 mice ranging from 8 to 10 weeks in age were randomly assigned to experimental groups (*N* = 5 treated, *N* = 4 control).

### Stereotaxic injections into striatum

Mice were all stereotactically injected into the right striatum (coordinates: anteroposterior: +0.6 mm, lateral: ±1.8 mm, ventral: −3.3 mm) with nanobiologicals in a final volume of 4 μl containing scProtein, LVV encoding FLEx-reporter and/or mammalian CRE plasmid. Control animals were injected with 4 μl containing scProtein, LVV encoding FLEx-reporter, and bacterial CRE plasmid, all at an infusion rate of 0.25 mL/min using a 10 mL Hamilton syringe. Five min after the infusion was completed, the needle was retracted 0.3 mm and allowed to remain in place for an additional 3 min prior to its complete removal from the mouse brain^35^.

### mouse tissue preparation for immunohistochemistry, bioluminescence, and RT-PCR

Mice were sacrificed with a 100–200 μl bolus of ketamine (17.5 mg/ml) and Xylazine (2.5 mg/ml) intraperitoneally followed by an intracardiac perfusion with 50 ml PBS. Brains were collected and frozen at −80 °C. Coronal sections of the striatum at 16 μm thickness were obtained using a cryostat (LEICA CM3050S, Leica Microsystems). Sections were alternately collected for immunohistochemistry, bioluminescence, and RT-PCR. Primers are listed in Supplementary Table 3.

### Statistical analysis and reproducibility

Data were analyzed using GraphPad Prism 9, version 9.1.0 (GraphPad Software Inc., La Jolla, CA). All statistical tests were two-sided and a p-value of less than 0.05 was considered statistically significant. Data were presented as the mean ± S.E.M. The statistical tests used are indicated in the figure legends. Multiple comparisons of significance between groups were performed using the Tukey procedure for ANOVA or Dunn’s multiple comparison test for Kruskal–Wallis, as indicated in the corresponding figure legends. The statistical analyses for uptake of EVs was modelled with non-linear regression using a one-phase association or one phase-decay equation. Graphical illustrations in figures were done in Adobe Illustrator 26.0.2.

### Reporting summary

Further information on research design is available in the Nature Research Reporting Summary linked to this article.

## Supplementary information

Supplementary Information

Reporting Summary

## Data Availability

The datasets to generate Figs. 1–5 of this manuscript, including the unprocessed gel of Fig. 4F are available on figshare: 10.6084/m9.figshare.19617147.v1.

## Materials availability

The materials generated during the current study are available from the corresponding author on reasonable request.
