# Supplementary material for: Exogenous loading of extracellular vesicles, virus-like particles, and lentiviral vectors with supercharged proteins
Source: Commun Biol. 2022 May 19;5:485. doi: 10.1038/s42003-022-03440-7 (PMC9120435; doi:10.1038/s42003-022-03440-7)
Supplement: Supplementary file 1 — Supplementary Information [file 42003_2022_3440_MOESM1_ESM.pdf]

Supplementary Information

# – FIGURES –

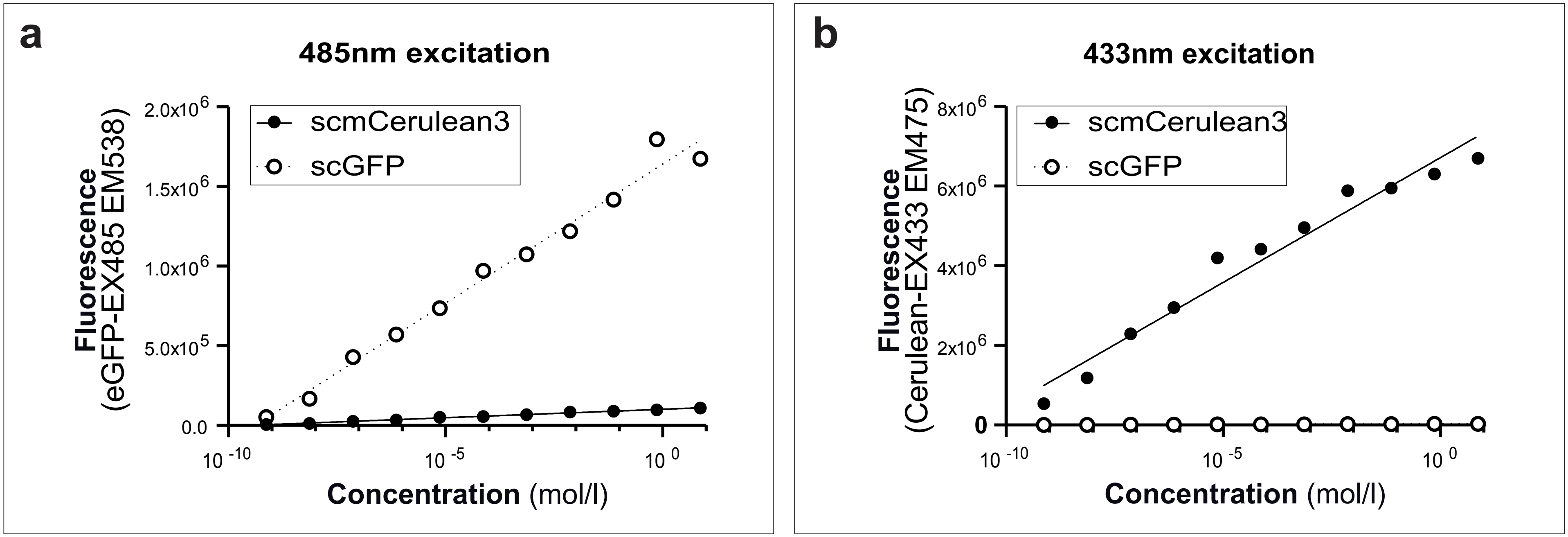

Figure S1. Mutagenesis of amino acids in functional site of scGFP alters fluorescent detection. (a) Graph showing the correlation between protein concentration and the fluorescence signal when excited at 485nm. **(b)** Graph showing the correlation between protein concentration and the fluorescence when excited at 433nm. The original scGFP protein is shown as open circles/dashed line while the newly generated scmCerulean3 is displayed as closed circles/solid line.

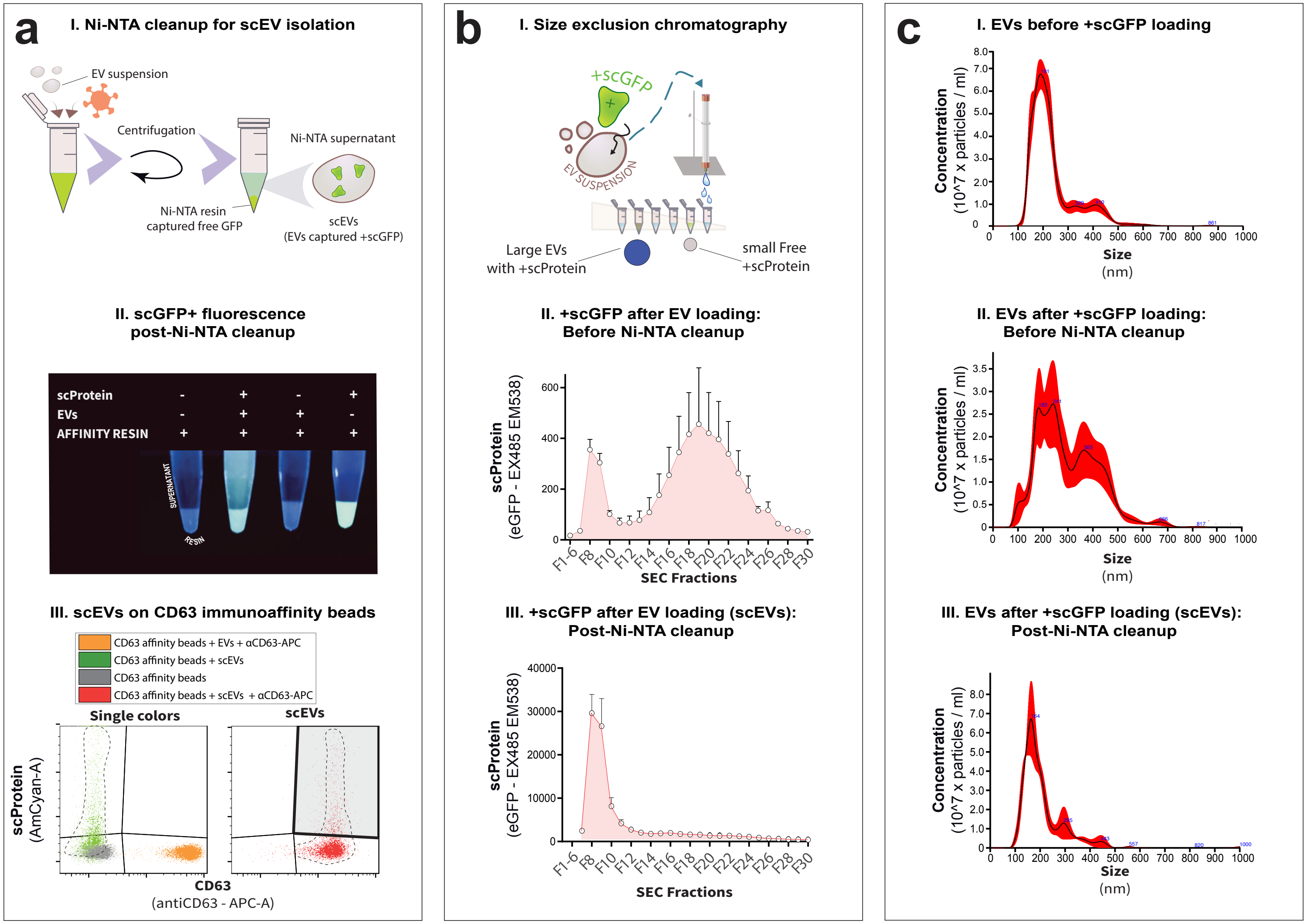

Figure S2. Bulk scEV characterization. (a) Purified EVs assemble with +scProtein to generate scEVs. **[I]** Schematic of how scEVs are isolated with Ni-NTA resin from a mixture of positive supercharged Protein (+scProtein) and purified EVs. **[II]** Green fluorescence under ultraviolet light of solutions that were exposed to Ni-NTA resin. The conditions from left to right are PBS (EV diluent), +scProtein and HEK293T EVs, HEK293T EVs alone, and +scProtein alone. On the bottom of the tube, we observe the pelleted Ni-NTA resin. **[III]** scEVs after Ni-NTA cleanup can be pulled down with αCD63 affinity beads co-immunoprecipitating +scProtein. On the left single fluorescent signals are shown, including αCD63 immunoaffinity beads alone (grey), with EVs and αCD63-APC (orange), and with HEK293T scEVs alone without αCD63-APC (green). On the right side α CD63 immunoaffinity beads with HEK293T scEVs and αCD63-APC (red) are shown. Dotted line represents +scProtein loaded EVs. Data acquired with flow cytometry. **(b)** Effect of Ni-NTA cleanup on scEV integrity demonstrated with size exclusion chromatography (SEC). **[I]** SEC aids in resolving unloaded +scProtein from scEVs in solutions, as shown in the schematic. Big particles such as HEK293T EVs are detected in the early SEC fractions, while small particles, such as free +scProtein come off in later SEC fractions. **[II]** +scProtein can be detected in both early and late SEC fractions in solutions with +scProtein mixed with EVs (n=3). **[III]** Post-Ni-NTA resin cleanup, we detected the +scProtein only in the early SEC fractions (n=3). Graphs represent green fluorescence derived from the recombinant +scProtein. **(c)** Effect of Ni-NTA cleanup on scEV integrity demonstrated with Nanosight analysis. **[I]** Nanosight analysis of HEK293T EVs alone (n=5), **[II]** HEK293T EVs and +scGFP (n=5), and **[III]** HEK-293T EVs and +scGFP after Ni-NTA resin cleanup (n=5). Data are presented with mean and SEM (error bars).

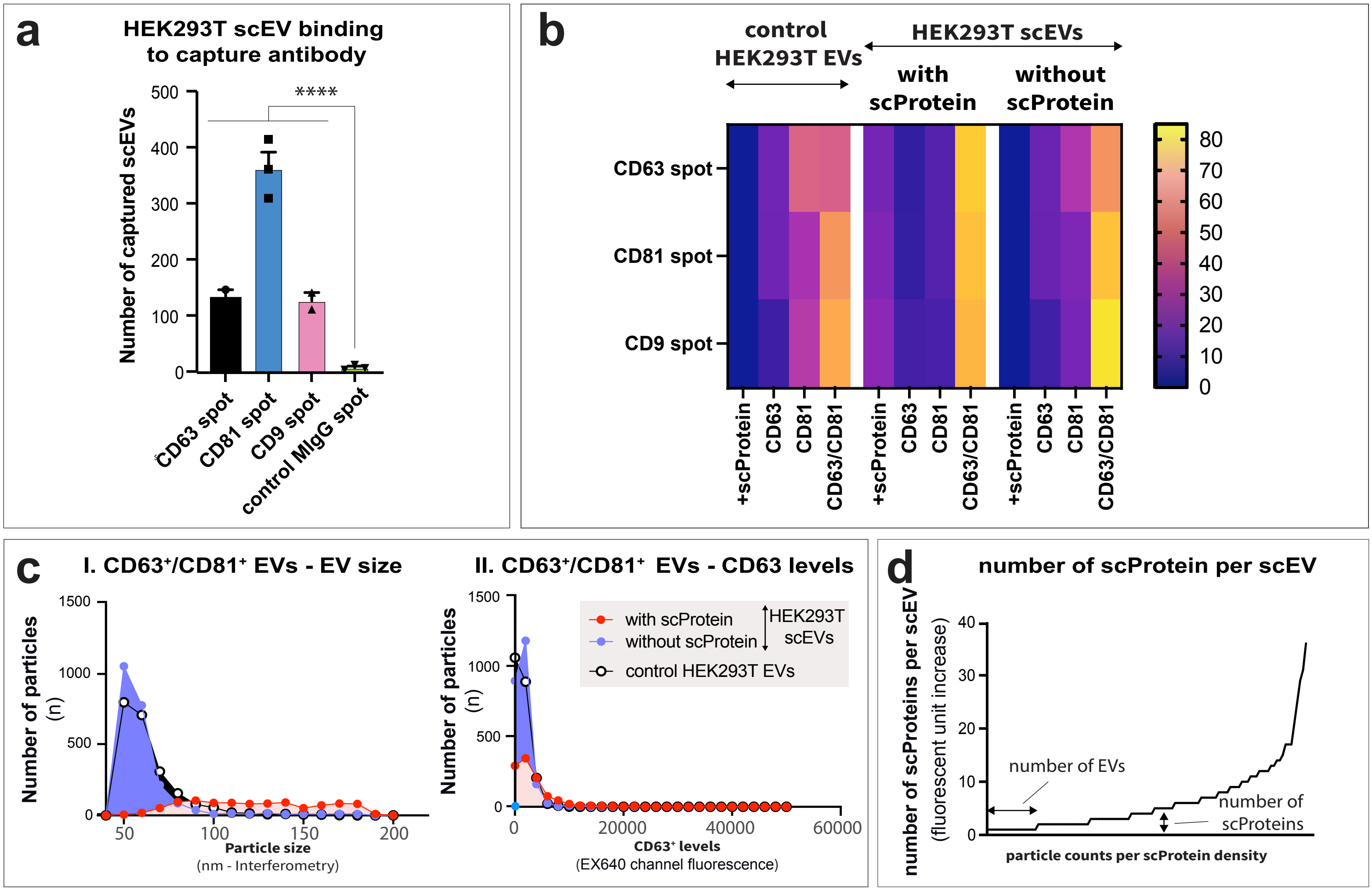

Figure S3. Single scEV characterization with Exoview. (a) Number of scEVs (after Ni-NTA cleanup) captured on an Exoview chip through αCD63, αCD81 or αCD9 binding spots compared to an MIgG control spot (n=3). **(b)** Heatmap of scEVs bound to either αCD63, αCD81 and αCD9 binding spots based on fluorescence of +scProtein and αCD63 and αCD81 detection antibodies. The samples tested are HEK293T EVs without (control HEK293T EVs) and with +scProtein association and Ni-NTA cleanup (HEK293T scEVs). In the latter sample we distinguished EVs that were positive for +scProtein (scEVs) and EVs that were negative for +scProtein. Data represents average colocalization percentage. **(c)** +scProteins were preferentially loaded into larger sized EVs in the CD63+CD81+ subpopulation. **[I]** We selected EVs captured by the αCD81 spot that were positive for both αCD63 and αCD81 and compared these hits among our different conditions (control HEK293T EVs, HEK293T scEVs loaded with scProtein, HEK293T scEVs not loaded scProtein). We compared the number of events with a certain particle size through interferometric measurements. **[II]** Graphs representing our three conditions (control HEK293T EVs, HEK293T scEVs loaded with scProtein, HEK293T scEVs not loaded with +scProtein) based on the fluorescence of the αCD63 detection antibody in the CD63+CD81+ EVs subpopulation. **(d)** +scProtein loading capacity of EVs. Y-axis represents the number of +scProteins per EV while the x-axis represents the number of EVs that were loaded with +scProtein cargo. Graph represents the +scProtein fluorescence increase per EV hit on the Exoview chip. Data are presented as the mean with SEM (error bars). Data analyzed with one-way ANOVA with Tukey's multiple-comparisons test. **** represents a p-value of <0.0001.

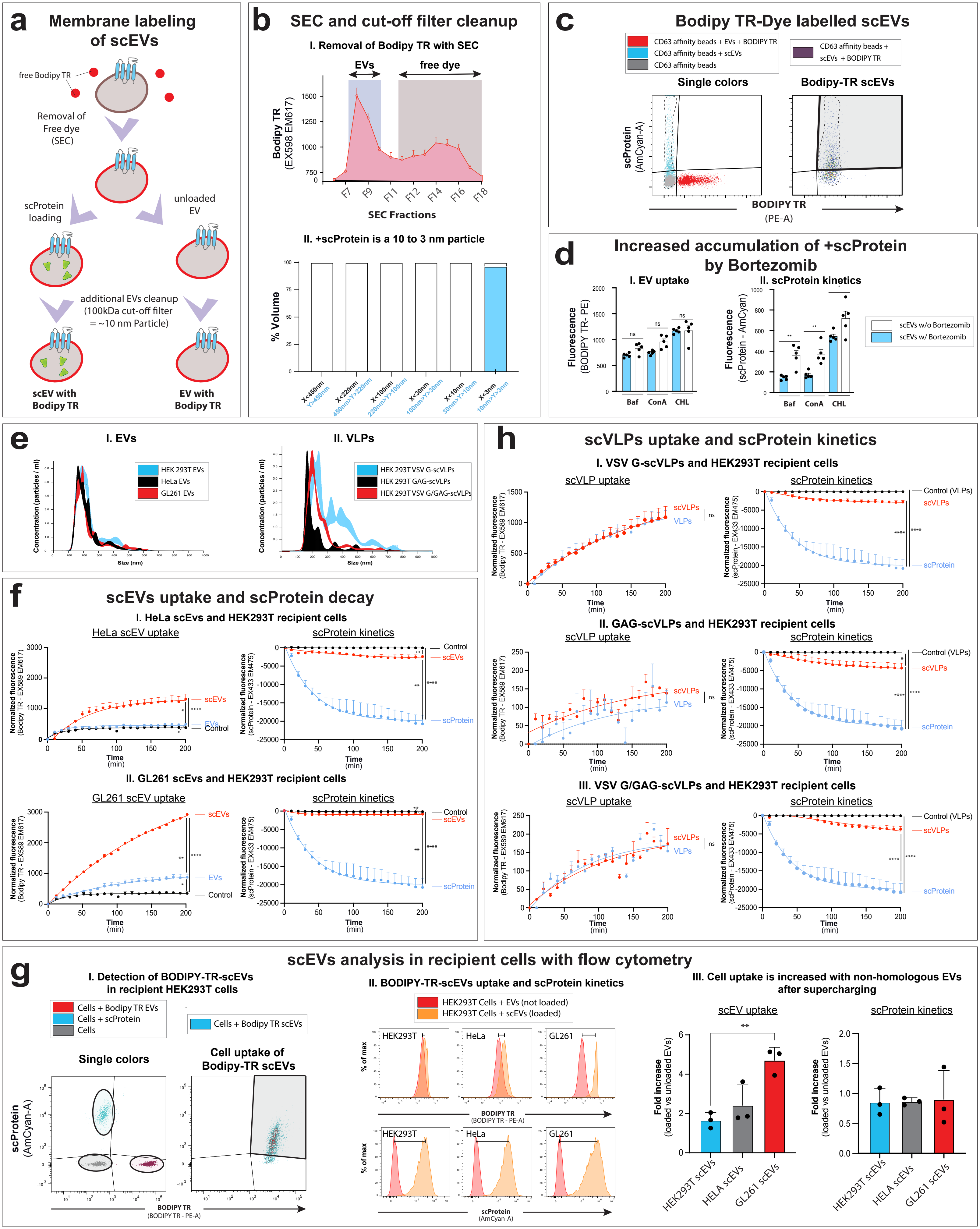

a
Membrane labeling of scEVs
free Bodipy TR
Removal of Free dye (SEC)
scProtein loading
unloaded EV
additional EVs cleanup (100kDa cut-off filter = ~10 nm Particle)
scEV with Bodipy TR
EV with Bodipy TR
b
SEC and cut-off filter cleanup
I. Removal of Bodipy TR with SEC
EVs
free dye
Bodipy TR (EX598 EM617)
SEC Fractions
F7 F9 F11 F12 F14 F16 F18
II. +scProtein is a 10 to 3 nm particle
% Volume
X<450nm Y>450nm
X<220nm 450nm>Y>220nm
X<100nm 220nm>Y>100nm
X<30nm 100nm>Y>30nm
X<10nm 30nm>Y>10nm
X<3nm 10nm>Y>3nm
c
Bodipy TR-Dye labelled scEVs
CD63 affinity beads + EVs + BODIPY TR
CD63 affinity beads + scEVs
CD63 affinity beads
CD63 affinity beads + scEVs + BODIPY TR
Single colors
Bodipy-TR scEVs
scProtein (AmCyan-A)
BODIPY TR (PE-A)
d
Increased accumulation of +scProtein by Bortezomib
I. EV uptake
II. scProtein kinetics
Fluorescence (BODIPY TR- PE)
Fluorescence (scProtein - AmCyan)
Baf ConA CHL
scEVs w/o Bortezomib
scEVs w/ Bortezomib
e
I. EVs
II. VLPs
Concentration (particles / ml)
Size (nm)
HEK 293T EVs
HeLa EVs
GL261 EVs
HEK 293T VSV G-scVLPs
HEK 293T GAG-scVLPs
HEK 293T VSV G/GAG-scVLPs
f
scEVs uptake and scProtein decay
I. HeLa scEvs and HEK293T recipient cells
HeLa scEV uptake
scProtein kinetics
Normalized fluorescence (Bodipy TR - EX589 EM617)
Normalized fluorescence (scProtein - EX433 EM475)
Time (min)
scEVs
EVs
Control
scProtein
II. GL261 scEvs and HEK293T recipient cells
GL261 scEV uptake
scProtein kinetics
h
scVLPs uptake and scProtein kinetics
I. VSV G-scVLPs and HEK293T recipient cells
scVLP uptake
scProtein kinetics
Control (VLPs)
scVLPs
VLPs
scProtein
II. GAG-scVLPs and HEK293T recipient cells
III. VSV G/GAG-scVLPs and HEK293T recipient cells
g
scEVs analysis in recipient cells with flow cytometry
I. Detection of BODIPY-TR-scEVs in recipient HEK293T cells
Cells + Bodipy TR EVs
Cells + scProtein
Cells
Cells + Bodipy TR scEVs
Single colors
Cell uptake of Bodipy-TR scEVs
scProtein (AmCyan-A)
BODIPY TR (BODIPY TR - PE-A)
II. BODIPY-TR-scEVs uptake and scProtein kinetics
HEK293T Cells + EVs (not loaded)
HEK293T Cells + scEVs (loaded)
HEK293T HeLa GL261
% of max
BODIPY TR (BODIPY TR - PE-A)
scProtein (AmCyan-A)
III. Cell uptake is increased with non-homologous EVs after supercharging
scEV uptake
scProtein kinetics
Fold increase (loaded vs unloaded EVs)
HEK293T scEVs
HELA scEVs
GL261 scEVs

Figure S4. Uptake of scBVs by cells in vitro. (a) Simplified schematic illustration of the scEV labeling procedure with Bodipy TR membrane dye. **(b)** Bodipy TR membrane dye cleanup procedures. **[I]** Size exclusion chromatography (SEC) eliminates free Bodipy TR dye. Profile of Bodipy TR fluorescence when a labeled EVs solution was applied to a SEC column. BODIPY TR labelled EVs (n=3) were retrieved in early SEC fractions, while in the later fractions free dye was observed. The blue band represents EV fractions, while brown band represents protein and free dye fractions. **[II]** Free scProtein contaminants were removed with a sized cut-off filter. Our scProtein has a size of 31 kDa, hence it is retained (represented in blue) in the 3nm/30kDa cut-off filter after serial filtration. In blue, the volume of liquid retained in the 3nm/30kDa filter is shown. Data is represented as percentage of volume, in which volume in both filter and flow through represent 100%. **(c)** Bodipy TR-labelled scEVs were bound to a αCD63-bead for detection with flow cytometry. Representative analysis of samples containing EVs with Bodipy TR dye without +scProtein (red), scEVs without Bodipy TR (blue), and no EVs (grey) on the left. A representative sample with scEVs and Bodipy TR can be seen on the right. The dotted line delineates the +scProtein loaded EV sample. **(d)** HEK293T scEV uptake with inhibitors. HEK293T cells were preincubated with v-ATPase blockers – 200 nM Bafilomycin A1 (Baf, n=5), 200 nM Concanamycin A (conA, n=5) or 10uM of the lysosomotropic Chloroquine (CHL, n=5) together with DMSO (white bars) with or without the 26S proteasome inhibitor - 5uM Bortezomib (blue bars) 1 h before the addition of HEK293T scEVs. After 24h scEV exposure, **[I]** Bodipy-TR and **[II]** +scProtein fluoresence was acquired with flow cytometry. **(e)** Nanosight analysis of **[I]** EVs derived from HEK293T (blue), Hela (black), and GL261 (red) cells show a similar profile and size distribution while **[II]** Virus-like particles (VLPs) derived from HEK293T with VSV G (blue), GAG (black), and VSV G/GAG (red) have different size distributions. **(f)** Uptake of scEVs of different origin by HEK293T recipient cells. **[I]** HeLa or **[II]** GL261 Bodipy TR labelled scEVs (n=3), +scProtein (n=3) and Bodipy-TR labelled EVs (control, n=3) were incubated with HEK293T cells. When scEVs were taken up by HEK293T cells, Bodipy TR signal increased in red fluorescence as a result of more internalization by HEK293T cells, while cyan fluorescence of +scProtein decreased over time due to degradation. Curves indicate nonlinear regression models; for [I]-Left: scEVs $R2=0.79$ and EVs $R2=0.87$; for [I]-Right: scEVs $R2=0.50$ and scProtein $R2=0.73$; for [II]-Left: scEVs $R2=0.99$ and EVs $R2=0.84$; for [II]-Right: scEVs $R2=0.08$ and scProtein $R2=0.73$. Bodipy TR (left) and scProtein (right) fluorescence was imaged every 10 min for 200 min. Each datapoint was normalized to the starting value. **(g)** scEVs and EVs were incubated with HEK293T cells for 24 h and fluorescence was acquired with flow cytometry. **[I]** Cells that were not exposed to EVs (grey, left dot plot) or scEVs (blue, left dot plot), and Bodipy TR labeled EVs (red, left dot plot) could be discriminated from cells that had taken up Bodipy TR labeled scEVs (blue, right dot plot). **[II]** After 24 h incubation of HEK293T cells with either Bodipy TR labelled HEK293T (n=3), HeLa (n=3) or GL261 (n=3) scEV (orange) or EVs (red), Bodipy TR (top) and scProtein fluorescence was compared with flow cytometry. **[III]** Bar graphs show that Bodipy TR fluorescent signal differed between EV donor cell type (left), while the scProtein fluorescent signal did not (right). **(h)** Bodipy TR-labelled VLPs were generated from HEK293T with **[I]** VSV G (n=3), **[II]** GAG (n=3), and **[III]** both VSVG/GAG (n=3) and incubated with HEK293T cells. Fluorescence was acquired as described in (f). Curves indicate nonlinear regression models; for [I]-Left: VLPs/scVLPs $R2 = 0.82$, [I]-Right: scVLPs $R2=0.70$, [II]-Left: VLPs/scVLPs $R2 = 0.49$, [II]-Right: scVLPs $R2=0.40$, [III]-Left: VLPs/scVLPs $R2 = 0.66$. [III]-Right: scVLPs $R2=0.51$. Data represent the median fluorescent signal of scEVs compared to the unloaded EV control. Data was analyzed with one-way ANOVA test. ****, ***, ** and * represent a p-value of <0.0001, <0.001, <0.01, and <0.05, respectively. R2 represents the statistical measure of how close the data are to the fitted regression line.

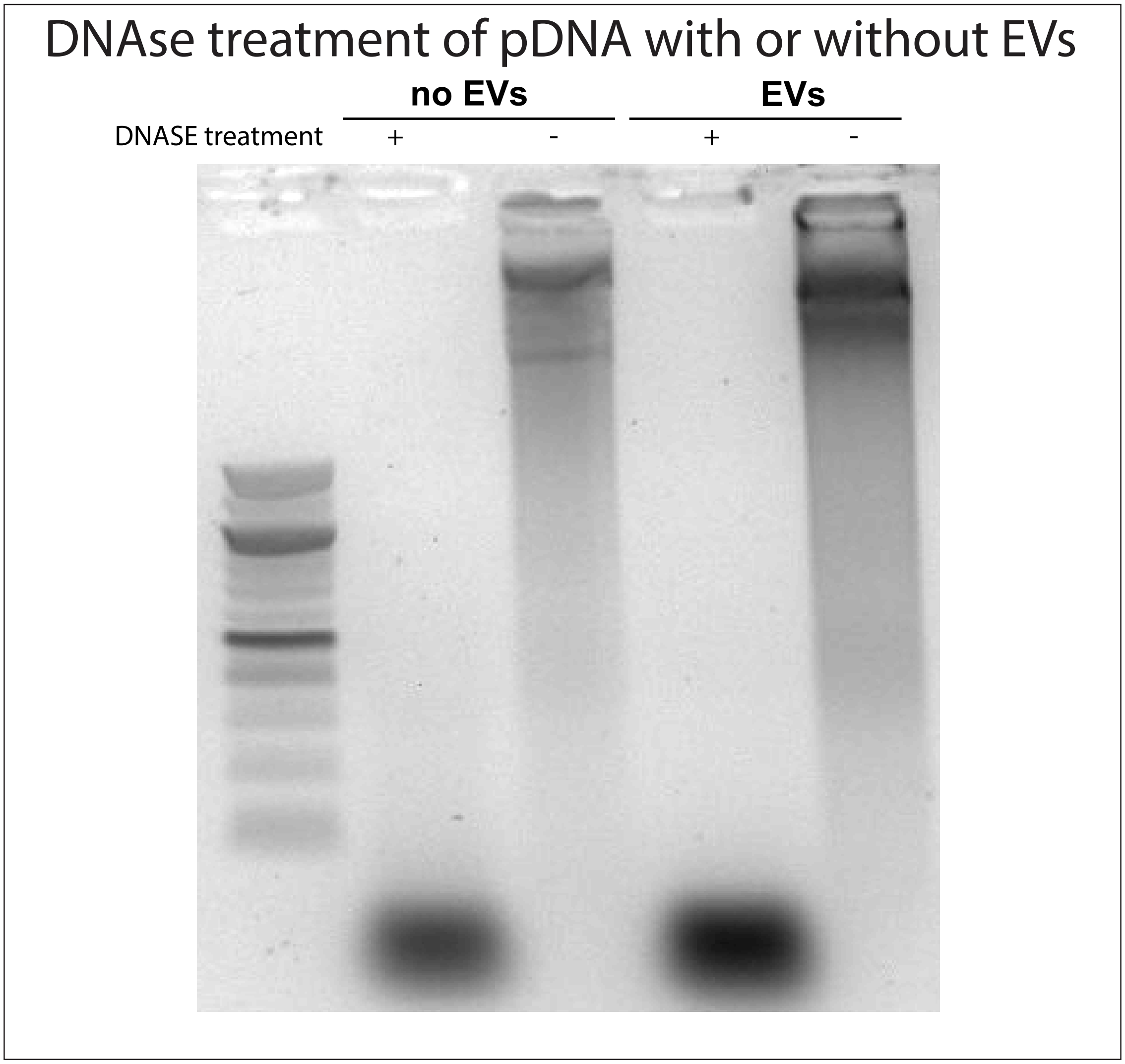

Figure S5. pDNA and DNAse I. DNAse I treatment degrades pDNA when it is not assembled in pDNA-scEVs. 1 μg pDNA suspensions without or with EVs were incubated for 15 min with DNAse I. Samples were loaded on an 0.75% agarose gel and visualized with GelRed Nucleic Acid dye.

Supplementary Information

# – METHODS –

TRANSMISSION ELECTRON MICROSCOPY (TEM). A 20ul HEK293T EV sample was directly applied on the grid. After 1 min absorbance onto the grid, the excess liquid is blotted off the film surface using a filter paper (Whatman). The grid is floated on a small drop (~5 µl) of staining solution (0.75% uranyl formate, 1% uranyl acetate or 1-2% PTA). After 20 seconds, the excess stain is blotted off and the sample is air dried briefly before it's examined in the TEM. Images were captured at the HMS electron microscopy core facility using Tecnai G2 Spirit Bio TWIN transmission electron microscope.

BULK BIOVESICLE NUMBER ESTIMATION WITH NANOPARTICLE TRACKING ANALYSIS. Number of BVs diluted in PBS was assayed using Nanoparticle Tracking Analysis Version 2.2 Build 0375 instrument (Nano Sight). Particles were measured for 60 s and the number of particles (30–800 nm) was determined using NTA Software 2.2. Samples were diluted 1:1000 in PBS prior to analysis. The following photographic conditions were used: frames processed (1498 of 1498 or 1499 of 1499); frames per second (24.97 or 24.98 f/s); calibration (190 nm/pixel); and detection threshold (6 or 7 multi).

PLASMID CONSTRUCTION. For production of bacteria expressing recombinant recombinant proteins construct were cloned into the pET28a vector (Addgene #85492). The plasmid was restricted with NcoI and XhoI and used as backbone for Gibson assembly (New England Biolabs) to insert the gBlock (IDT) expression cassettes of scProteins (+scGFP, -scGFP, GFP, +scCER, +NLS-scCER, see amino acid sequences in supplementary data). Each assembly reaction contained approximately 100 ng insert and 50 ng expression vector and was incubated at 50°C for 30 min - 4 hrs following the manufacturer's protocol. After the assembly reaction, the reaction mix was transformed into NEB 5-alpha competent *E. coli* strain (New England Biolabs) or One Shot®TOP10 Competent Cells (ThermoFisher). After overnight growth at 37°C on Kanamycin 50 µg/mL (Sigma) containing agar plates [10 ml Bacto agar (Sigma) with LB medium in a 60 mm dish], single colonies were selected and grown with Kanamycin 50 µg/mL (Sigma) containing LB broth (Sigma). Single colony suspensions containing respective plasmid with insert were extracted using a QIAprep Spin Miniprep Kit (Qiagen). To confirm correct insertion, a restriction digest was performed, and fragments electrophoresed in a 1.5% agarose gel and stained with GelRed (Biotium). Images were acquired under UV light using Azure Biosystems c300 Image. When correct profiles were detected, complete plasmid sequencing using next-generation sequencing technology (MGH CCIB DNA Core) was performed to validate plasmid integrity. Vectors generated following this procedure and used for this manuscript are summarized in Supplementary Table 1.

scPROTEIN PURIFICATION. The protein purification protocol is an adaptation of Thompson et al., 2008. Frozen pellets were thawed and resuspended in 50 mL PBS with 2 M NaCl, 20 mM imidazole, pH 7.5, with one tablet of EDTA-free Complete Protease Inhibitor (Roche). The resuspended pellets were divided into two fractions and lysed by sonication in a Sonic Dismembrator 550 (Fisher Scientific) for 5 min. Cell debris was removed by centrifugation at 4000 × g for 10 min. The supernatant was transferred to a new 50 mL conical tube and 1 mL of settled Ni-NTA agarose resin (Qiagen) was added to the bacterial lysate and incubated at 4°C for 30–45 min with a HulaMixer™. Then the supernatant and the Ni-NTA resin were transferred to a column. The packed resin was rinsed first with 20 mL PBS and 2 M NaCl, then with 15 mL of PBS, 2 M NaCl, and 20 mM imidazole, and finally eluted with 2 mL PBS, 2 M NaCl, and 500 mM imidazole. To remove the imidazole from the protein solution it was dialyzed against 1 L PBS at 4°C for 1 hr, and subsequently dialyzed overnight with 2 L fresh PBS buffer. Proteins were quantified via fluorescence and BSA protein (Pierce™, ThermoFisher) assay. Purity and protein size was confirmed by SDS-PAGE and nitrocellulose blot transfer. Protein bands were visualized with Pierce™ Reversible Protein Stain Kit for Nitrocellulose (Thermo Scientific). Aliquots were stored at −80°C.

FLOW CYTOMETRY. Flow cytometric analysis was performed on the Beckman SORP 5 Laser BD Fortessa Flow Cytometer in the MGH core facility. Forward and side scatter signals were used to distinguish live cells from polystyrene beads. Proper gating was performed to identify positive fluorescent signals compared to non-stained or single stained controls.

LENTIVIRAL VECTOR (LVV) PRODUCTION AND CELL TRANSDUCTION. LVVs were produced in HEK293T cells with a three-plasmid system, following Addgene recommendations. Briefly, cells were seeded and 24 hrs later, transfected with psPAX2 (#12260) and pMD2.G (#12259) packaging plasmids and the transgene of interest flanked by LTRs. Six hrs after transfection, cells were rinsed with PBS and media was replaced. Lentiviral isolation was performed 72 hrs later by ultracentrifugation at 70,000 x g and the pellet was re-suspended in 1% BSA in PBS[36]. The viral particle content was evaluated by assessing HIV-1 p24 antigen levels by ELISA (Retro Tek, Gentaur, Paris, France). Concentrated viral stocks were stored at −80°C until use. LVVs were used for generating stable cell lines after selection by either antibiotic resistance or flow sorting. HEK293T and HELA cells were transduced 24 hrs after plating with LVVs (400 ng of P24 HIV antigen per 200,000 cells). Twenty-four hours later, the medium was replaced with DMEM media with antibiotics and FBS, and cells were cultured and expanded under standard conditions. Stable cell lines were obtained and mCherry or GFP fluorescence was monitored at every passage.

IMMUNOHISTOCHEMISTRY. Sections were fixed with 4% paraformaldehyde for 10 min and incubated with blocking solution [0.1% Triton X-100 containing 10% normal goat serum (Sigma-Aldrich) in PBS] and then incubated overnight at 4°C in blocking solution with primary antibody: rabbit anti-RFP antibody (1:250Invitrogen, polyclonal). Sections were rinsed with PBS 5 times and incubated for 2 hr at room temperature with the secondary antibody: goat anti-rabbit TRITC 594 (1:1000) diluted in blocking solution. The sections were washed and mounted in VECTASHIELD® Antifade Mounting Medium (Vector Labs Cat# H-1000) on gelatin-coated slides. Immunoreactivity of mouse sections was visualized and analyzed in Keyence BZ-X810 All-in-one Fluorescence imaging microscope.

DNA AND RNA EXTRACTIONS. The DNeasy Blood & Tissue Kit (Qiagen) was used for genomic DNA extraction from cells.QIAprep Spin Miniprep Kit (Qiagen) and EndoFree MaxiPlasmid Kits (Qiagen) were used for plasmid extraction from bacteria. RNA was extracted using the miRNeasy Mini Kit (Qiagen), according to manufacturer's protocol. The RNA concentration and integrity (RIN score) were determined using the Nanodrop (ThermoFischer Scientific) and Agilent 2100 Bioanalyzer Pico-chips (Agilent Technologies), following manufacturer's protocol.

CONFOCAL MICROSCOPY. 10,000 cells were seeded on a 35 mm optical Petri dish (Thermofisher) and incubated with approx. 10^6 scEVs for 5 days at 37ºC. Prior to imaging, LysoTracker Red DND-99 (Invitrogen) was added to the media to a final working concentration of 50nM for 1h. The cells were washed twice with fresh media prior to imaging on a Zeiss LSM710 Laser Scanning Confocal of the MGH Cancer Center Translational Imaging Core. Of note, Lysotracker Red DND-99 was incubated under similar conditions with isolated EVs and did not stain our scEVs. To detect nuclear translocation 10,000 cells were seeded on an optical Petri dish (theromofisher) and incubated with 10^6 scEVs for 5days at 37ºC. Cells were fixed with paraformaldehyde for 15 min at RT, washed twice with PBS and incubated with a AF594-Phalloidin (Invitrogen) according to manufacturer instructions. After twice washing with PBS, Dapi was provided through VECTASHIELD® Antifade Mounting Medium (Vector Labs Cat# H-1000). ImageJ version 2.1.0/1.53c was used for analysis.

Supplementary Information

# – SUPPLEMENTARY NOTE 1 –

Amino acid sequence of recombinant proteins used in Breyne *et al.*. Proteins were either mutated in functional site (indicated by blue highlight) or in solvent exposed surface (indicated by purple and yellow highlight for Arg and Lys, respectively). To generate a negatively charged protein Asp and Glu were incorporated (indicated by red and green highlight, respectively).

Legend:

AA mutated to change emission/excitation wavelength
AA mutated to alter net charge of protein
Positively charged AA added to scaffold: Arginine, Lysine
Negatively charged AA added to scaffold: Aspartic acid, Glutamic acid

Recombinant proteins used in manuscript:

AA sequence eGFP

MVSKGEELFTGVVPILVELDGDVNGHKFSVRGEGEGDATNGKLTLKFICTTGKLPVPW
PTLVTTLTYGVQCFSRYPDHMKRHDFFKSAMPEGYVQERTISFKDDGTYKTRAEVKFE
GDTLVNRIELKGIDFKEDGNILGHKLEYNFNSHNVYITADKQKNGIKANFKIRHNVEDGS
VQLADHYQQNTPIGDGPVLLPDNHYLSTQSVLSKDPNEKRDHMVLLEFVTAAGITHGM
DELYK

AA sequence positive supercharged eGFP

MASKGERLFRGKVPILVELKGDVNGHKFSVRGKGKGDATRGKLTLKFICTTGKLPVPW
PTLVTTLTYGVQCFSRYPKHMKRHDFFKSAMPKGYVQERTISFKKDGKYKTRAEVKFE
GRTLVNRIKLKGRDFKEKGNILGHKLRYNFNSHKVYITADKRKNGIKAKFKIRHNVKDG
SVQLADHYQQNTPIGRGPVLLPRNHYLSTRSKLSKDPKEKRDHMVLLEFVTAAGIKHG
RDERYK

AA sequence negative supercharged eGFP

MSKGEELFDGVVPILVELDGDVNGHEFSVRGEGEGDATEGELTLKFICTTGELPVPWP
TLVTTLTYGVQCFSDYPDHMDQHDFFKSAMPEGYVQERTISFKDDGTYKTRAEVKFE
GDTLVNRIELKGIDFKEDGNILGHKLEYNFNSHDVYITADKQENGIKAEFEIRHNVEDGS
VQLADHYQQNTPIGDGPVLLPDDHYLSTESALSKDPNEDRDHMVLLEFVTAAGIDHGM
DELYK

AA sequence of supercharged mCerulean3

MVSKGERLFRGKVPILVELKGDVNGHKFSVRGKGKGDATRGKLTLKFICTTGKLPVPW
PTLVTTLSWGVQCFARYPKHMKRHDFFKSAMPKGYVQERTISFKKDGKYKTRAEVKF
EGRTLVNRIKLKGRDFKEKGNILGHKLRYNAIHGKVYITADKRKNGIKAKFGLNCNVKD

GSVQLADHYQQNTPIGRGPVLLPRNHYLSTRSKLSKDPKEKRDHMVLLEFVTAAGIKL
GRDERYK

Supplementary Information

# – SUPPLEMENTARY TABLES –

Supplementary Table 1. Plasmids used in this manuscript:

Transgene	source
sfGFP	Addgene #85492
-scGFP	Addgene #62936
+scGFP	Addgene #62937
+scmCerulean3	This paper
+scmCerulean3-NLS	This paper
CD63-nanoluc-flag-mCardinal	This paper and Rufino-Ramos *et al.* 2022
FLEx-GFP-floxed nanoluc	This paper
mCherry LVV plasmid	Addgene #78534
mCre plasmid	This paper
bCre plasmid	This paper

Supplementary Table 2. Number of Arginine and Lysine residues incorporated in recombinant proteins used in this manuscript:

Charged AA	+scGFP	eGFP	-scGFP	+scmCerulean3
R	20	8	6	19
K	36	20	13	35

Supplementary Table 3. Primers used in this manuscript:

Primer name	Primer sequence	Details
Forw_Nanoluc_PP3	CACTGGTAATCGACGGGGTT	Unfloxed Combination
Forw_EF1A_PP2	GGGGAGGGGTTTTATGCGAT	
Forw_EF1A_PP2	GGGGAGGGGTTTTATGCGAT	Floxed 1 Combination
Rev_Nanoluc_PP2	AACACGGCGATGCCTTCATA	
Forw_Nanoluc_PP3	CACTGGTAATCGACGGGGTT	Floxed 2 Combination
PP2_WPRE ver	GTTGCGTCAGCAAACACAGT	
PP2_WPRE Fwrd	CGCTATGTGGATACGCTGCT	

PP2_WPRE ver	GTTGCGTCAGCAAACACAGT	WPRE
Ai9 CAG Fwrd	GCAACGTGCTGGTTATTGTG	Ai9 Unfloxed Combination
REV_UNFLOXED_Ai9_185bp	TGCAAGCTTTCATTTATTCATCGC	
Ai9 CAG Fwrd	GCAACGTGCTGGTTATTGTG	Ai9 Floxed Combination
REV_Ai9_U1140_F269bp	TTTGATGACCTCCTCGCCCT	
