# Supplementary material for: Exogenous loading of extracellular vesicles, virus-like particles, and lentiviral vectors with supercharged proteins
Source: Commun Biol. 2022 May 19;5:485. doi: 10.1038/s42003-022-03440-7 (PMC9120435; doi:10.1038/s42003-022-03440-7)
Supplement: Supplementary file 2 — Reporting Summary [file 42003_2022_3440_MOESM2_ESM.pdf]

nature portfolio

Corresponding author(s): Koen Breyne

Last updated by author(s): Mar 30, 2022

Reporting Summary

Nature Portfolio wishes to improve the reproducibility of the work that we publish. This form provides structure for consistency and transparency in reporting. For further information on Nature Portfolio policies, see our Editorial Policies and the Editorial Policy Checklist.

Statistics

For all statistical analyses, confirm that the following items are present in the figure legend, table legend, main text, or Methods section.

n/a	Confirmed	
☐	☒	The exact sample size (*n*) for each experimental group/condition, given as a discrete number and unit of measurement
☐	☒	A statement on whether measurements were taken from distinct samples or whether the same sample was measured repeatedly
☐	☒	The statistical test(s) used AND whether they are one- or two-sided<br>*Only common tests should be described solely by name; describe more complex techniques in the Methods section.*
☒	☐	A description of all covariates tested
☐	☒	A description of any assumptions or corrections, such as tests of normality and adjustment for multiple comparisons
☐	☒	A full description of the statistical parameters including central tendency (e.g. means) or other basic estimates (e.g. regression coefficient) AND variation (e.g. standard deviation) or associated estimates of uncertainty (e.g. confidence intervals)
☐	☒	For null hypothesis testing, the test statistic (e.g. *F*, *t*, *r*) with confidence intervals, effect sizes, degrees of freedom and *P* value noted<br>*Give P values as exact values whenever suitable.*
☒	☐	For Bayesian analysis, information on the choice of priors and Markov chain Monte Carlo settings
☒	☐	For hierarchical and complex designs, identification of the appropriate level for tests and full reporting of outcomes
☐	☒	Estimates of effect sizes (e.g. Cohen's *d*, Pearson's *r*), indicating how they were calculated

Our web collection on statistics for biologists contains articles on many of the points above.

Software and code

Policy information about availability of computer code

Data collection	N/A
Data analysis	N/A

For manuscripts utilizing custom algorithms or software that are central to the research but not yet described in published literature, software must be made available to editors and reviewers. We strongly encourage code deposition in a community repository (e.g. GitHub). See the Nature Portfolio guidelines for submitting code & software for further information.

Data

Policy information about availability of data

All manuscripts must include a data availability statement. This statement should provide the following information, where applicable:

- Accession codes, unique identifiers, or web links for publicly available datasets
- A description of any restrictions on data availability
- For clinical datasets or third party data, please ensure that the statement adheres to our policy

The datasets generated during and/or analyzed during the current study are available from the corresponding author on reasonable request.

Field-specific reporting

Please select the one below that is the best fit for your research. If you are not sure, read the appropriate sections before making your selection.

☒ Life sciences ☐ Behavioural & social sciences ☐ Ecological, evolutionary & environmental sciences

For a reference copy of the document with all sections, see nature.com/documents/nr-reporting-summary-flat.pdf

Life sciences study design

All studies must disclose on these points even when the disclosure is negative.

Sample size	No sample-size determination calculation was performed.
Data exclusions	No data was excluded from the dataset.
Replication	Two to three replications were performed to generate the data represented in this manuscript.
Randomization	Ai9 mice were randomly taken from litters of Ai9 breeders.
Blinding	Analysis of samples was performed/repeated by different investigators without information about the outcome of the experiment.

Reporting for specific materials, systems and methods

We require information from authors about some types of materials, experimental systems and methods used in many studies. Here, indicate whether each material, system or method listed is relevant to your study. If you are not sure if a list item applies to your research, read the appropriate section before selecting a response.

Materials & experimental systems

n/a	Involved in the study	
☐	☒	Antibodies
☐	☒	Eukaryotic cell lines
☒	☐	Palaeontology and archaeology
☐	☒	Animals and other organisms
☒	☐	Human research participants
☒	☐	Clinical data
☒	☐	Dual use research of concern

Methods

n/a	Involved in the study	
☒	☐	ChIP-seq
☐	☒	Flow cytometry
☒	☐	MRI-based neuroimaging

Antibodies

Antibodies used	anti-CD63, anti-CD9, anti-RFP, anti-rabbit-TRITC
Validation	anti-CD63 and anti-CD9 for exoview analysis were provided and verified by the manufacturer. Binding to Isotype control spot was used as a negative control. anti-CD63-APC (MEM-259) was used for flow cytometry and was validated against human CD63. A sample without EVs but with only beads was used as a staining control. Primary anti-RFP (R10367, 1:250) and secondary anti-TRITC (goat anti-rabbit, Invitrogen, 1:1000) for immunohistochemistry was validated on wild type mouse brains as a negative control.

Eukaryotic cell lines

Policy information about cell lines

Cell line source(s)	HEK293T, GL261, HeLa
Authentication	Cells were purchased from ATCC.
Mycoplasma contamination	Cells were monthly tested for mycoplasma contamination (Mycoplasma PCR Detection Kit, abm G238) and found negative.
Commonly misidentified lines (See ICLAC register)	N/A

Animals and other organisms

Policy information about studies involving animals; ARRIVE guidelines recommended for reporting animal research

Laboratory animals	Male and female Ai9 mice ranging from 8-10 weeks in age were randomly assigned to experimental groups.
Wild animals	N/A
Field-collected samples	N/A
Ethics oversight	All animal experiments were conducted under the oversight of the MGB Institution Animal Care and Use Committee.

Note that full information on the approval of the study protocol must also be provided in the manuscript.

Flow Cytometry

Plots

Confirm that:

☒ The axis labels state the marker and fluorochrome used (e.g. CD4-FITC).

☒ The axis scales are clearly visible. Include numbers along axes only for bottom left plot of group (a 'group' is an analysis of identical markers).

☒ All plots are contour plots with outliers or pseudocolor plots.

☒ A numerical value for number of cells or percentage (with statistics) is provided.

Methodology

Sample preparation	Cells were trypsinized, washed with PBS, 40um filtered to single cells, and kept on ice prior to measurement. Immunocapture beads were washed 3 times prior to Fc blocking and antibody staining.
Instrument	Flow cytometric analysis was performed on the Beckman SORP 5 Laser BD Fortessa Flow Cytometer in the MGH core facility.
Software	FlowJo software was used for the analysis of our flow cytometric data.
Cell population abundance	Cultured cells were used, no mixed cell types were measured for flow cytometry.
Gating strategy	Single stainings were used to gate for double positive populations. This is exemplified in figure S2-A3 and S4 - H1.

☒ Tick this box to confirm that a figure exemplifying the gating strategy is provided in the Supplementary Information.
